# LRP2-mediated regulation of ferroptosis through the Wnt/β-catenin–GPX4 axis in colorectal cancer liver metastasis and chemoresistance

**DOI:** 10.1038/s41420-026-03161-4

**Published:** 2026-05-29

**Authors:** Shasha Zhao, Limei Sun, Jing Xia, Sen Yan, Baohua Zhang, Buyou Lu, Nan Huang, Fenyong Sun, Fuming Shen

**Affiliations:** 1 Department of Clinical Laboratory, Shanghai Tenth People’s Hospital, School of Medicine, Tongji University, Shanghai, 200092 China; 2 Department of Clinical Pharmacy, School of Basic Medicine and Clinical Pharmacy, China Pharmaceutical University, Nanjing, 211198 China; 3 Department of Pharmacy, Shanghai Tenth People’s Hospital, School of Medicine, Tongji University, Shanghai, 200092 China

**Keywords:** Metastasis, Gastrointestinal cancer

## Abstract

Colorectal cancer liver metastasis (CRLM) is a major contributor to cancer mortality, and therapeutic efficacy is often limited by chemoresistance. Dysregulated lipid metabolism has been implicated in tumor progression, yet the function of low-density lipoprotein receptor–related protein 2 (LRP2) in CRLM and oxaliplatin (Oxa) resistance has not been defined. We integrated bioinformatics analyses of TCGA and GSE131418 datasets with in vitro and in vivo experiments to investigate the role of LRP2 in CRLM. Untargeted metabolomics, ferroptosis assays, and mechanistic studies were employed to characterize the Wnt/β-catenin–GPX4 axis. LRP2 was markedly upregulated in CRLM and associated with poor survival. Silencing LRP2 inhibited colorectal cancer (CRC) cell proliferation, migration, and liver metastasis, while enhancing sensitivity to Oxa. LRP2 depletion induced ferroptosis, which was rescued by ferroptosis inhibition. Mechanistically, LRP2 activated Wnt/β-catenin signaling, driving GPX4 expression through TCF1-mediated transcription. LRP2 promotes CRLM and Oxa resistance by repressing ferroptosis via the Wnt/β-catenin–TCF1–GPX4 pathway. Targeting this signaling cascade may provide a therapeutic strategy for metastatic CRC.

## Introduction

Colorectal cancer (CRC) ranks as the third most common malignancy worldwide, accounting for nearly 10% of cancer-related deaths [1]. Metastasis is the primary determinant of mortality: while localized disease has a five-year survival rate exceeding 90%, this falls to ~50% in regionally advanced cases and ~10% once distant metastases occur [2]. The liver is the most frequent metastatic site. Approximately one-quarter of patients present with synchronous hepatic metastases at diagnosis, and another quarter develop metachronous metastases following resection [3]. Without treatment, survival after liver metastasis averages only 5–10 months, with negligible five-year survival [3]. Despite advances in surgery and chemotherapy, CRLM continues to confer a dismal prognosis [4]. Understanding the molecular drivers of metastasis and resistance is essential to improve therapeutic outcomes.

Aberrant lipid metabolism has emerged as a hallmark of malignancy, influencing tumor initiation, progression, and therapy response [5–7]. Lipid metabolism in cancer cells is shaped both by oncogenic signaling and by interactions with the tumor microenvironment, which provides diverse factors including cytokines, nucleic acids, and lipids. In turn, metabolic alterations can remodel signaling pathways and affect surrounding cells through secreted lipid mediators [7]. To identify lipid metabolism–related genes relevant to CRLM, we performed integrative bioinformatics analyses using lipid metabolism gene sets from the Molecular Signatures Database (MSigDB) and transcriptomic data from The Cancer Genome Atlas (TCGA). This analysis highlighted low-density lipoprotein receptor–related protein 2 (LRP2) as a top candidate associated with CRLM.

LRP2 (also known as megalin) is a multifunctional endocytic receptor belonging to the low-density lipoprotein receptor (LDLR) family, which regulates cholesterol uptake and cellular homeostasis [8]. LDLR family members have been implicated in the pathogenesis of several cancers, including colorectal, prostate, lung, breast, and liver cancers [9, 10]. LRP2 mediates clathrin-dependent internalization of a wide range of ligands, including lipoproteins, steroid hormones, and retinoids [11]. It plays critical roles in embryonic development, renal function, and cardiovascular and nervous system biology [12–16]. However, its role in tumor biology, particularly in CRLM, remains poorly defined.

In this study, we combined bioinformatics, metabolomics, and functional experiments to characterize the biological role of LRP2 in CRLM and to elucidate its mechanistic link to chemoresistance.

## Results

### High LRP2 expression predicts poor prognosis in CRC

**Fig. 1:**
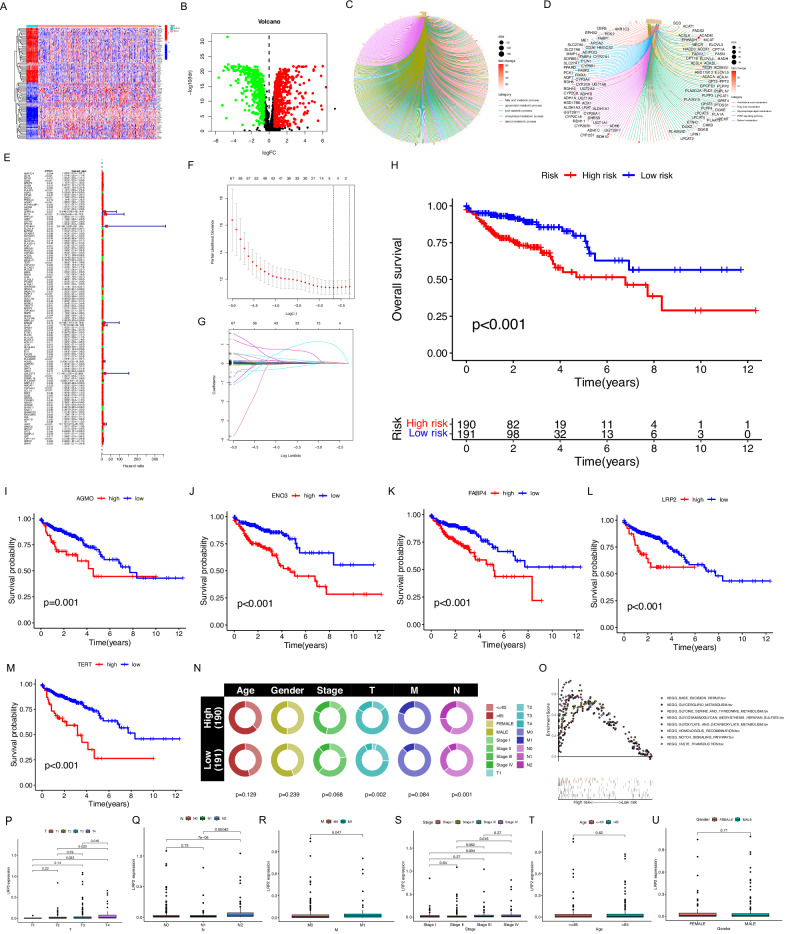
High LRP2 expression predicts poor prognosis in CRC. Heatmap (**A**) and volcano plot (**B**) of 1 005 differentially expressed lipid metabolism genes in TCGA CRC cohort. Blue/green colors indicate low expression of genes, and red colors represent high expression of genes. GO (**C**) and KEGG (**D**) enrichment analyses of differentially expressed genes. **E** Forest plot of 113 prognostic genes identified by Cox Proportional Hazards Regression analysis among the 1005 differentially expressed genes. Green indicates a hazard ratio for this gene of less than 1, while red signifies a hazard ratio greater than 1. **F**, **G** LASSO Cox regression model identifying five prognostic genes (ENO3, AGMO, TERT, FABP4, LRP2) based on these 113 prognostic genes. **H** The Kaplan–Meier plots demonstrated the overall survival rate of patients in high- and low-risk groups within the TCGA dataset. **I**–**M** Separate Kaplan–Meier plots were generated for the survival analysis of each of the five model genes. **N** Circle map displayed the clinical characteristic differences between high- and low-risk groups of patients within the TCGA dataset. **O** Representative pathways with significant differences between the high-risk score and low-risk score groups enriched by GSEA analysis. **P**–**U** LRP2 expression among patients grouped based on distinct clinical features.

To identify lipid metabolism–associated genes relevant to CRLM, we analyzed TCGA CRC datasets using MSigDB lipid gene sets. A total of 1005 lipid-related genes were differentially expressed, including 613 upregulated and 392 downregulated (Fig. 1A, B). GO and KEGG analyses revealed enrichment in fatty acid metabolism, lipid catabolism, and glycerophospholipid pathways (Fig. 1C, D).

Among these, 113 genes correlated with overall survival in univariate Cox regression (Fig. 1E). LASSO regression yielded a five-gene signature (*ENO3, AGMO, TERT, FABP4, LRP2*) with prognostic value based on these 113 genes (Fig. 1F, G), as expressed by the following formula:

$${Riskscore}=(0.0213* {ENO}3)+(0.0177* {AGMO})+(0.0692* {TERT})+(0.0002* {FABP}4)+(1.0122* {LRP}2)$$

Kaplan–Meier analysis confirmed that patients with higher risk scores had significantly reduced survival (Fig. 1H). Survival analysis for individual genes showed that elevated expression predicted worse outcomes (Fig. 1I–M). Risk scores also correlated with tumor stage (T, N; Fig. 1N). GSEA demonstrated that pathways differing significantly between the high- and low-risk groups were enriched in base excision repair, glycerolipid metabolism, and glycine–serine–threonine metabolism (Fig. 1O).

Within this model, *LRP2* carried the greatest weight (1.0122). Further analysis confirmed that high LRP2 expression was associated with poor prognosis and advanced TNM stage (Fig. 1P-S), but not with age or gender (Fig. 1T, U). Notably, *LRP2* was uniquely correlated with distant metastasis (M stage), whereas the other four genes were not (Supplementary Fig. 1). These findings suggested that *LRP2* is a potential driver of liver metastasis in CRC.

### LRP2 expression is elevated in CRLM

**Fig. 2:**
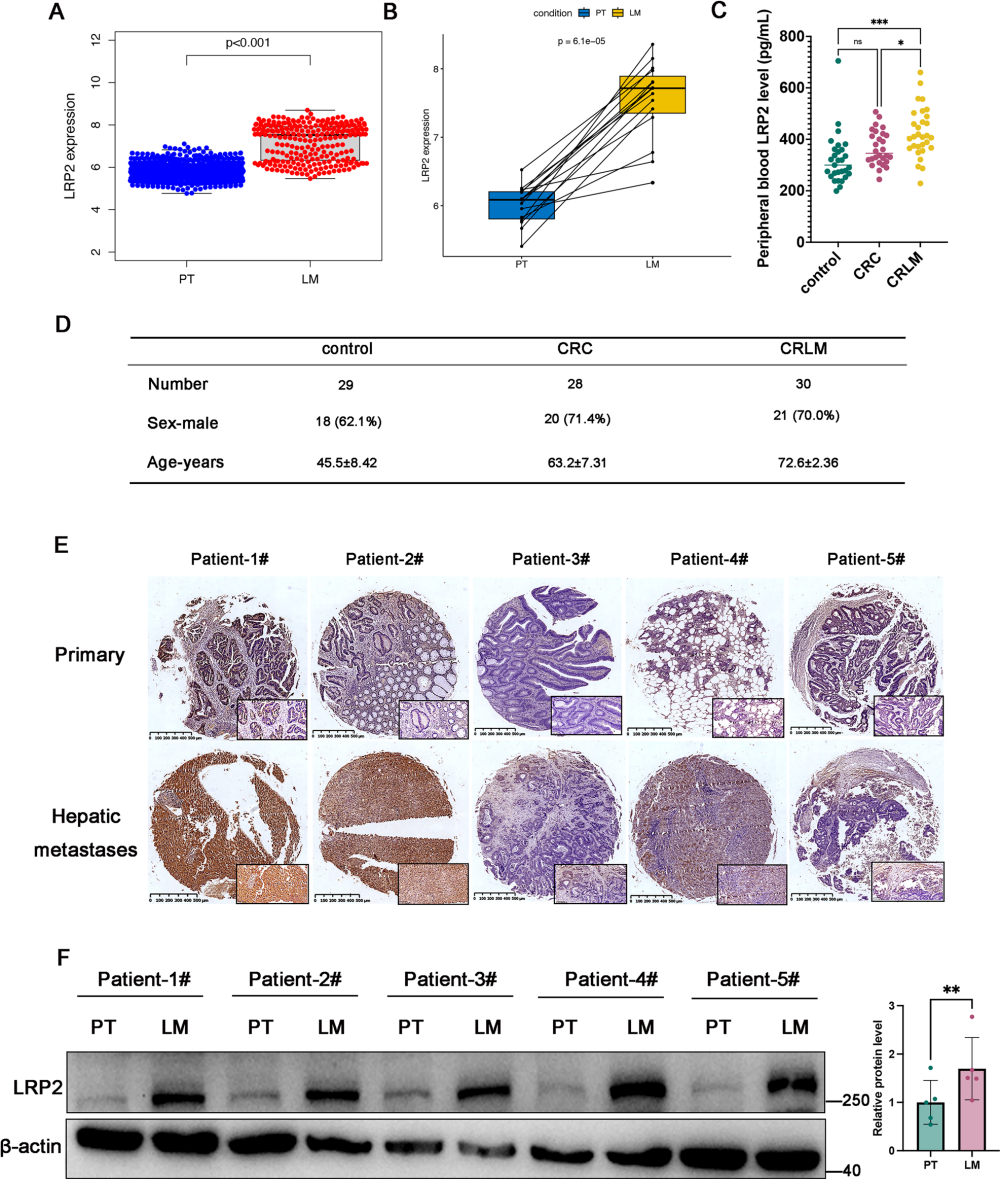
LRP2 expression is elevated in CRLM. LRP2 expression in PT and LM tissues (**A**), as well as in 15 paired samples of PT and LM tissues (**B**), of CRC patients from GSE131418 dataset. LRP2 levels in peripheral blood (**C**), and clinical data (**D**), of healthy volunteers, CRC and CRLM patients. **E** Images of IHC staining for LRP2 in PT tissues and the paired LM tissues from CRC patients (*n* = 5, scale bar = 500 µm). **F** LRP2 protein expression in PT tissues and the paired LM tissues from CRC patients (*n* = 5).

Analysis of GSE131418 showed significantly higher LRP2 expression in liver metastases (LM) compared with primary tumors (PT) (Fig. 2A). In 15 paired primary–metastasis samples, LRP2 was consistently upregulated in LM tissues (Fig. 2B). Peripheral blood plasma levels of LRP2 were also increased in CRLM patients relative to CRC patients and healthy controls (Fig. 2C, D). IHC and Western blotting further confirmed higher LRP2 expression in hepatic metastases than in matched primary tumors (Fig. 2E, F).

### LRP2 deficiency suppresses CRC cell proliferation and metastasis

**Fig. 3:**
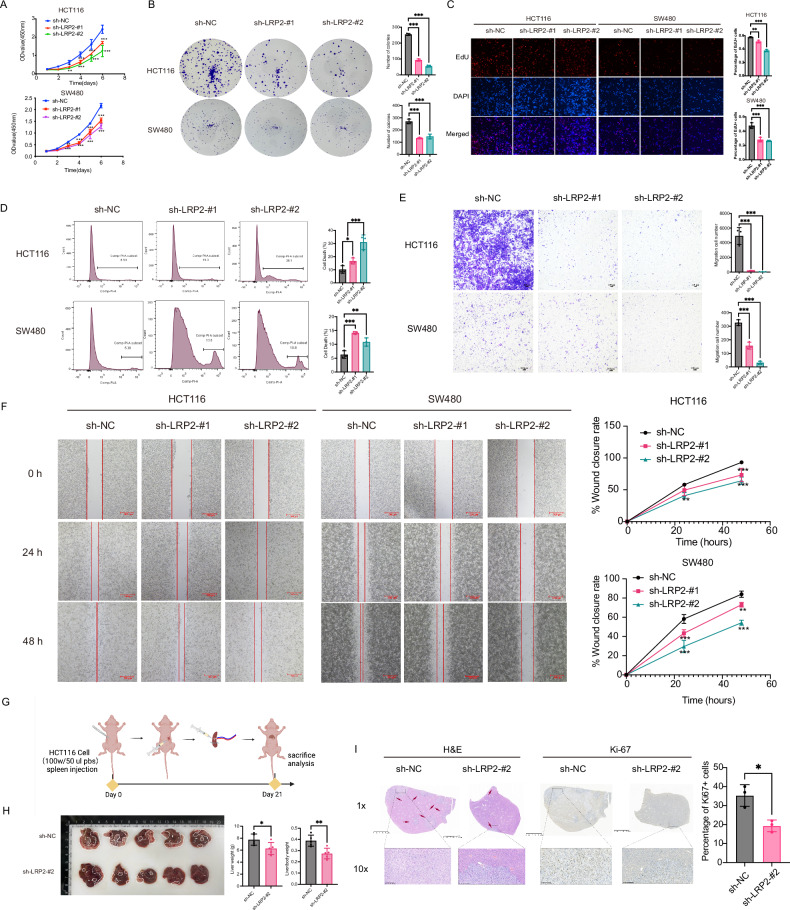
LRP2 deficiency suppresses CRC cell proliferation and metastasis. CCK8 (**A**), clone formation (**B**), and EdU staining assays (**C**) analysis of the proliferation of CRC cells upon the indicated transfections (*n* = 3). **D** Propidium iodide staining and cell death analysis of CRC cells upon the indicated transfections using flow cytometry (*n* = 3). **E** Transwell analysis for CRC cells migration and invasion after LRP2 knockdown (*n* = 3, scale bar = 100 μm). **F** Representative images of the wound healing assay in CRC cells at 0, 24 and 48 h after scratching (*n* = 3, scale bar = 500 μm). **G** Study design of liver metastasis xenograft nude-mouse models with CRC. **H**, **I** Gross appearance and weights of livers induced by splenic injected with sh-NC or sh-LRP2-#2 HCT116 cells in nude mice (the area circled out by the white dashed represents the tumor lesion) (**H**), and representative IHC staining of H&E and Ki67 in these livers (**I**) (*n* = 5, scale bar = 100 μm).

Stable LRP2 knockdown (sh-LRP2) was established in HCT116 and SW480 cells (Supplementary Fig. 2A, B). CCK-8, colony formation, and EdU assays demonstrated that LRP2 silencing significantly reduced proliferation (Fig. 3A–C). Flow cytometry revealed increased cell death in LRP2-deficient cells (Fig. 3D). Transwell and wound-healing assays showed impaired migration in sh-LRP2 groups of both HCT116 and SW480 cells (Fig. 3E, F). These findings indicated that LRP2 deficiency inhibited the metastasis and growth of CRC cells in vitro.

To assess whether LRP2 promote CRC liver metastasis in vivo, a xenograft model was generated by injecting sh-LRP2 or negative control (sh-NC) HCT116 cells into the spleen (Fig. 3G). The results showed that LRP2 knockdown formed fewer and smaller liver metastases in xenografted mice, with reduced Ki-67 staining (Fig. 3H, I). These data indicated that LRP2 promotes CRC cell growth and metastasis in vivo.

### LRP2 depletion enhances oxaliplatin sensitivity

**Fig. 4:**
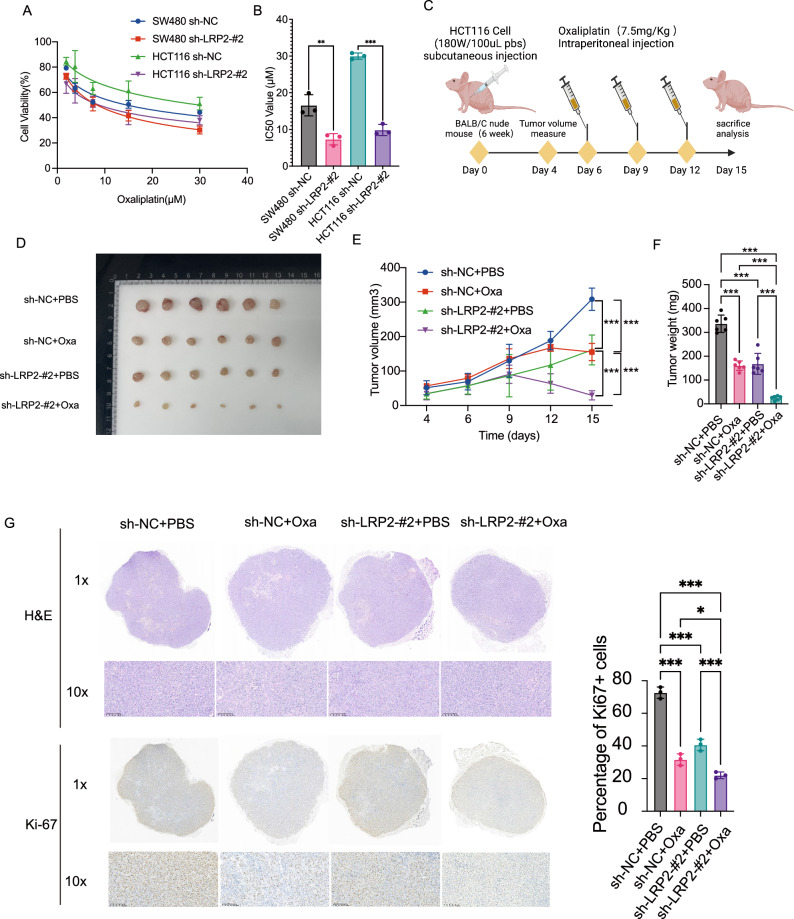
LRP2 depletion enhances oxaliplatin sensitivity. Cell viability assay in SW480 and HCT116 cells transfected with sh-LRP2-#2 or sh-NC treated with increasing concentrations of oxa (1.875–30 μM) (**A**), and the calculated IC50 values (*n* = 3) (**B**). **C** Schematic of the HCT116 cells in vivo xenograft and oxaliplatin treatment experiment. Gross appearance of subcutaneous tumors induced by subcutaneously injected with sh-NC or sh-LRP2-#2 HCT116 cells, followed by oxaliplatin or PBS treatment (intraperitoneal injection) in nude mice (**D**), tumor growth curves (**E**), quantitative analysis of tumor weights at endpoint (**F**), and representative IHC staining of H&E and Ki67 in these tumors (scale bar = 200 μm) (**G**) (***P < 0.001, *n* = 6 per group).

Given its association with poor prognosis, and that chemoresistance remains the major obstacle in treating CRLM, we assessed whether LRP2 influences oxaliplatin (Oxa) response. In vitro, LRP2 knockdown reduced Oxa IC50 by ~3-fold in HCT116 cells (from 29.98 ± 0.85 μM to 9.84 ± 1.50 μM, *P* < 0.001) and ~2-fold in SW480 cells (from 16.58 ± 2.89 μM to 7.314 ± 1.55 μM, *P* < 0.01) (Fig. 4A, B).

In subcutaneous xenografts (Fig. 4C), Oxa treatment markedly suppressed tumor growth in sh-LRP2 tumors compared with controls (Fig. 4D–F). IHC analysis further confirmed lower Ki-67 expression in LRP2-depleted tumors (Fig. 4G), suggesting decreased proliferative activity. Thus, LRP2 loss sensitized CRC cells to Oxa.

### LRP2 suppression remodels lipid metabolism and activates ferroptosis

**Fig. 5:**
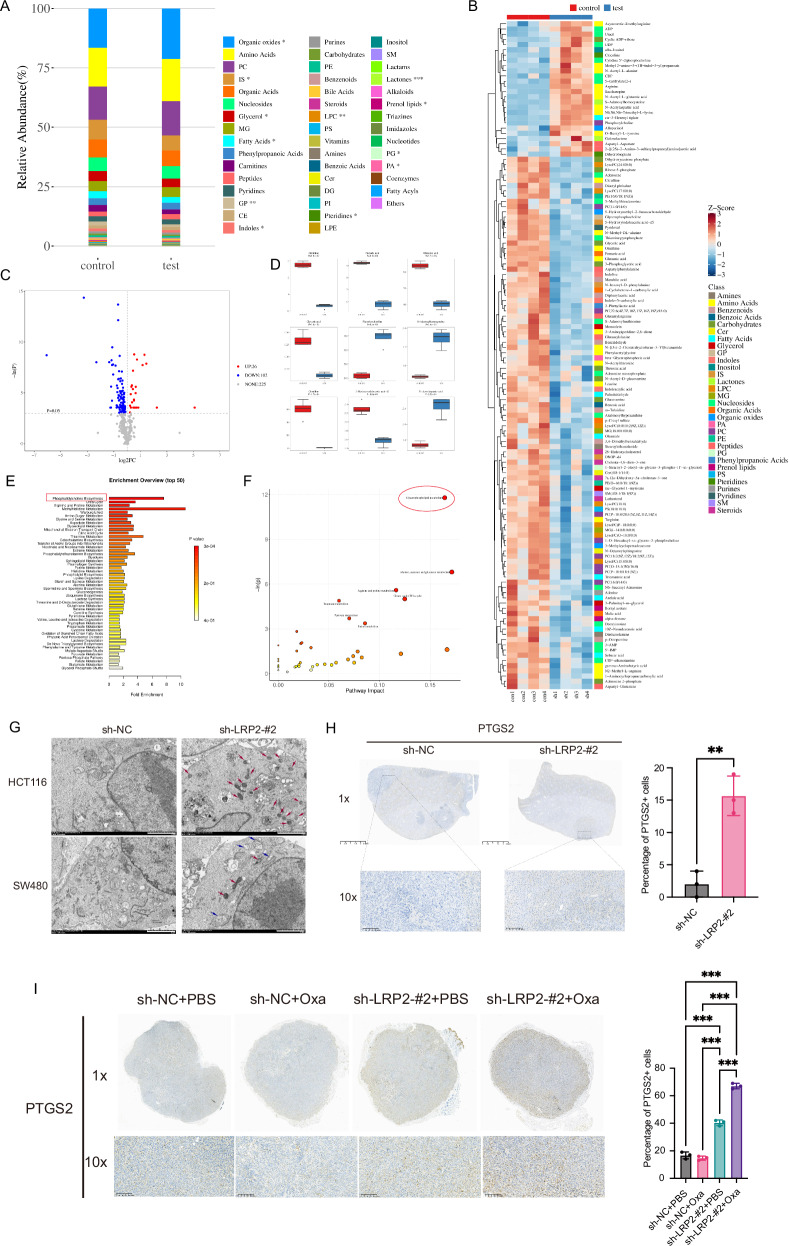
LRP2 suppression remodels lipid metabolism and activates ferroptosis. **A**–**F** Untargeted metabolomics profiling of sh-LRP2 vs sh-NC HCT116 cells (*n* = 4). Stacked bar chart of the relative abundance of each metabolite classes in different groups (**A**). Heat maps (**B**) and volcanic maps (**C**) of the differential metabolites in sh-LRP2 groups compared with sh-NC groups, in which red highlight are increased in test and blue highlight are decreased in test, threshold value for differential metabolites selection is: *P* < 0.05 and |log2FC|> = 0. Boxplot of the top 9 differential metabolites ordered by *P* value (**D**). Pathway Enrichment Analysis Barplot Using Pathway-associated metabolite sets (SMPDB) (**E**), and Pathway Analysis Bubble Plot by hsa set (**F**). **G** Transmission electron microscopy images of sh-NC and sh-LRP2 CRC cells. Red arrows indicate shrunken mitochondria with increased density, while blue arrows point to mitochondria with cristae disappearance (*n* = 3, Scale bars: 2.0 μm). IHC quantification of PTGS2 in livers of sh-LRP2 groups and sh-NC groups from liver metastasis xenografts models (*n* = 5, scale bar = 100μm) (**H**), and in subcutaneous tumors of sh-LRP2 and sh-NC groups from subcutaneous tumor models that treated by oxa or PBS (*n* = 6, scale bar = 100 μm) (**I**).

Untargeted metabolomics was conducted to explore the mechanistic of LRP2 in CRLM pathogenesis. The results demonstrated that LRP2 depletion drives metabolic reprogramming in colorectal cancer cells, yielding 225 significantly altered metabolites in sh-LRP2 HCT116 cells (26 upregulated, 199 downregulated; Fig. 5A–D). Pathway enrichment highlighted glycerophospholipid metabolism as the most affected pathway (Fig. 5E, F).

Given the established association between dysregulated glycerophospholipid metabolism and ferroptosis, we conducted preliminary assessments of ferroptotic responses in CRC cell lines and xenograft models. Transmission electron microscopy showed characteristic ferroptotic morphology in LRP2-deficient cells, including mitochondrial shrinkage and cristae loss (Fig. 5G). In liver metastasis models, IHC demonstrated increased PTGS2 (a ferroptosis marker) in sh-LRP2 tumors (Fig. 5H). Strikingly, subcutaneous tumor models demonstrated even higher PTGS2 expression when LRP2 knockdown combined with Oxa treatment (Fig. 5I). Together, these findings indicate that LRP2 depletion promotes ferroptosis.

### LRP2 knockdown suppresses metastasis and growth and enhances Oxa sensitivity in CRC cells via ferroptosis induction

Building upon our previous findings that LRP2 knockdown suppressed proliferation, inhibited migration, enhanced Oxa sensitivity and induced ferroptosis in CRC cells, we next examined whether these phenotypic changes were mechanistically driven by ferroptosis activation.

**Fig. 6:**
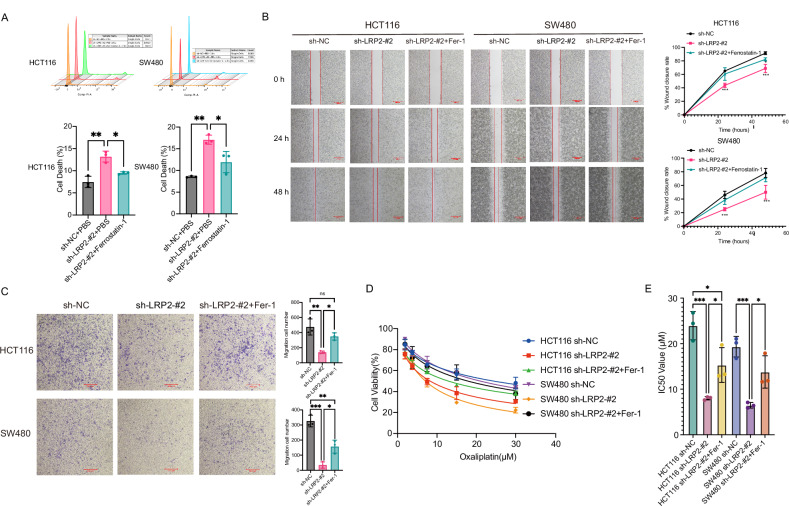
LRP2 knockdown suppresses metastasis and growth and enhances Oxa sensitivity in CRC cells via ferroptosis induction. **A** CRC cells transfected with either sh-NC or sh-LRP2, with or without treatment with Ferrostatin-1, was quantified using propidium iodide (PI) staining followed by flow cytometric analysis (*n* = 3). **B** Representative images of the wound healing assay in sh-NC or sh-LRP2 CRC cells, with or without treatment with Ferrostatin-1, at 0, 24 and 48 h after scratching (*n* = 3, scale bar = 500 μm). **C** Transwell analysis for sh-NC or sh-LRP2 CRC cells migration and invasion with or without Ferrostatin-1 treatment (*n* = 3, scale bar = 500 μm). **D**, **E** Cell viability assay in HCT116 and SW480 cells transfected with sh-LRP2 or sh-NC, with or without treatment with Ferrostatin-1, treated with increasing concentrations of oxa (1.875–30 μM) (**D**), and the calculated IC50 values (*n* = 3) (**E**).

To determine whether ferroptosis accounted for these effects, we treated cells with the ferroptosis inhibitor Ferrostatin-1 (Fer-1). Flow cytometric analysis of PI-stained cells revealed that inhibition of ferroptosis rescued sh-LRP2-induced cell death (Fig. 6A). Wound healing and transwell assays demonstrated partially restored migration in sh-LRP2 cells following ferroptosis inhibition (Fig. 6B, C). Inhibition of ferroptosis also increased Oxa IC50 values in both HCT116 (from 8.00 ± 0.36 μM to 15.31 ± 3.82 μM, *P* < 0.05) and SW480 cells (from 6.47 ± 0.60 μM to 13.79 ± 3.55 μM, *P* < 0.05) (Fig. 6D, E). These results demonstrate that ferroptosis is central to the antitumor and chemosensitizing effects of LRP2 depletion.

### LRP2 regulates ferroptosis through the Wnt/β-catenin–GPX4 axis

**Fig. 7:**
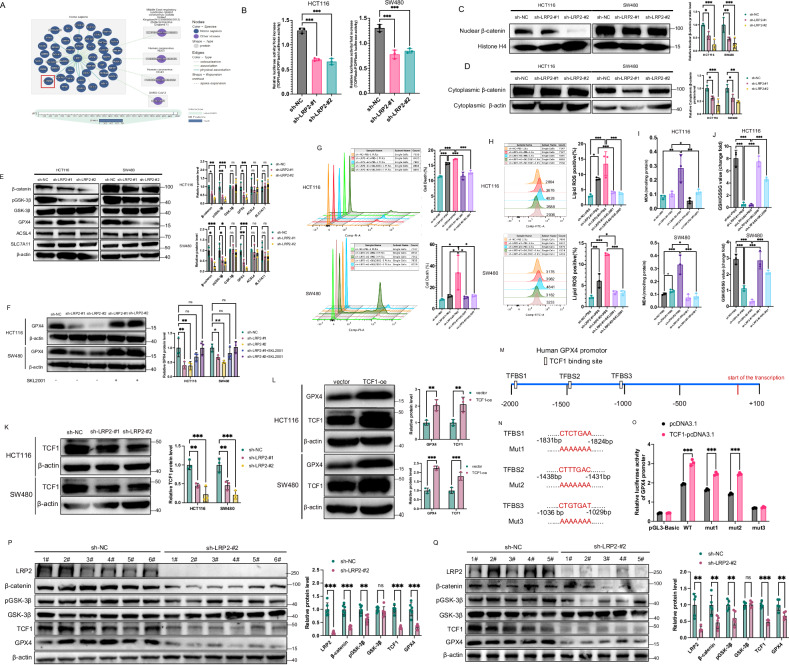
LRP2 regulates ferroptosis through the Wnt/β-catenin–TCF1-GPX4 signaling. **A** Predicted LRP2 protein interaction network. **B** TOPFlash/FOPFlash reporter activity in sh-NC vs sh-LRP2 cells. (*n* = 3). Analysis of nuclear β-catenin protein (**C**) and cytoplasmic β-catenin protein (**D**) level in sh-NC or sh-LRP2 CRC cells. **E** Expression levels of β-catenin, GSK-3β, pGSK-3β and key ferroptosis-related proteins in sh-NC and sh-LRP2 CRC cells. **F** Expression of GPX4 in sh-NC or sh-LRP2 CRC cells with or without SKL2001 addition. **G** Cell death in sh-NC or sh-LRP2 CRC cells with or without SKL2001 addition was measured via PI staining coupled with flow cytometry (*n* = 3). **H** Lipid ROS generation in sh-NC or sh-LRP2 CRC cells with or without SKL2001 addition was measured by C11-BODIPY581/591 staining coupled with flow cytometer (*n* = 3). MDA production (**I**) and relative ratio of GSH/GSSG (**J**) in sh-NC or sh-LRP2 CRC cells with or without SKL2001 addition (*n* = 3). **K** Analysis of TCF1 protein expression in sh-NC or sh-LRP2 CRC cells. **L** Protein expression of GPX4 in CRC cells overexpressing TCF1. **M** The three possible TCF1 binding sites in human GPX4 promoter. **N** The TCF1 binding site in human GPX4 promoter and the corresponding base mutation. **O** Transcriptional activity of GPX4 in HCT116 cells co-transfected with pcDNA3.1 or TCF1-pcDNA3.1 and luciferase reporter plasmids containing the wild-type GPX4 promoter or the indicated mutant GPX4 promoter fragments measured by the luciferase reporter system. **P** WB analysis of LRP2, β-catenin, pGSK-3β, GSK-3β, TCF1, and GPX4 expression in subcutaneous xenograft tumors derived from sh-NC or sh-LRP2-#2 CRC cells. **Q** WB analysis of the same signaling and ferroptosis-related proteins in liver metastatic lesions formed by sh-NC or sh-LRP2-#2 CRC cells. β-actin was used as a loading control, whereas pGSK-3β was quantified and normalized to total GSK-3β. Each lane represents tumor tissue from an individual mouse.

To investigate the specific molecular mechanism of LRP2 regulates ferroptosis in CRC cells, protein interaction analysis (IntAct database system, https://www.ebi.ac.uk/intact/home) was employed and identified β-catenin (CTNNB1) as a potential LRP2 partner (Fig. 7A). The subsequent dual-luciferase assays confirmed that LRP2 knockdown reduced Wnt signaling activity (*P* < 0.001; Fig. 7B), while WB analysis showed decraesed β-catenin accumulation in both nuclear and cytoplasmic compartments (Fig. 7C, D). Consistently, LRP2 deficiency led to a reduction in the expression of both β-catenin and phosphorylated GSK-3β at Ser9 (pGSK-3β^Ser9), whereas the total GSK-3β protein level remained relatively unchanged (Fig. 7E), further supporting the suppression of Wnt signaling.

Importantly, knockdown of LRP2 resulted in a marked downregulation of GPX4, a central regulator of ferroptosis (Fig. 7E). In contrast, the expression levels of two additional ferroptosis-related proteins (ACSL4 and SLC7A11) did not exhibit consistent evidence of regulation under the same conditions (Fig. 7E). Notably, pharmacological activation of Wnt signaling using the Wnt agonist SKL2001 effectively restored GPX4 expression in LRP2-deficient cells (Fig. 7F). Consistently, inhibition of Wnt signaling with DKK1 suppressed GPX4 at both mRNA and protein levels, which was reversed by SKL2001(Supplementary Fig. 3A–C).

Functionally, SKL2001 attenuated ferroptosis markers induced by LRP2 loss, including increased cell death (Fig. 7G), accumulation of reactive oxygen species (ROS) and lipid peroxidation product malondialdehyde (MDA) (Fig. 7H, I), and decreased GSH/GSSG ratio (Fig. 7J). These data demonstrate that LRP2 regulates ferroptosis via the Wnt/β-catenin–GPX4 axis.

### TCF1 directly drives GPX4 transcription

Following the activation of wnt/β-catenin signal pathway, β-catenin translocate into the nucleus and associates with the transcription factors TCF/LEF to promote the transcription of Wnt-target genes. Among Wnt pathway transcription factors, both TCF1 and TCF4 were reduced after LRP2 knockdown (Fig. 7K, Supplementary Fig. 4A). While TCF4 regulation of GPX4 has been reported in other cancer [17], we focus on investigating TCF1-dependent regulation of GPX4 in CRC.

As shown in Fig. 7L, overexpression of TCF1 increased GPX4 expression, establishing GPX4 as a functional downstream target of TCF1-mediated transcriptional control. Then, bioinformatic analysis identified three TCF1-binding motifs in the GPX4 promoter (Fig. 7M). The three TCF1-binding motifs and the corresponding base mutation were shown in Fig. 7N. Dual-luciferase reporter assays showed that mutations in motifs 1 and 2 preserved TCF1-driven GPX4 activation, while mutation in motif 3 (…CTGTGAT…) abolished this phenomenon (Fig. 7O). These findings established GPX4 as a direct transcriptional target of TCF1.

To further validate these findings, we examined the expression of key proteins involved in the Wnt signaling pathway and GPX4 in both subcutaneous xenograft tumors and liver metastatic lesions from mice. As shown in Fig. 7P, Q, knockdown of LRP2 resulted in a marked reduction in Wnt pathway–associated protein expression, accompanied by a concomitant decrease in GPX4 levels. These in vivo results further support that LRP2 deficiency suppresses Wnt signaling and GPX4 expression.

## Discussion

Although members of the LDLR family have been implicated in cancer biology, the role of LRP2 in CRLM and Oxa resistance had not been investigated. Our study provides the first comprehensive evidence that LRP2 is markedly upregulated in CRLM, correlates with advanced TNM stage, and predicts poor survival. Functional experiments demonstrated that LRP2 depletion suppresses tumor growth and enhances chemosensitivity. Untargeted metabolomics revealed profound lipid metabolic remodeling upon LRP2 silencing, with glycerophospholipid pathways most affected. Since these pathways are closely linked to ferroptosis susceptibility, we examined whether LRP2 influences ferroptotic signaling. Indeed, LRP2 loss induced ferroptotic morphology, increased ROS and lipid peroxidation, and upregulated PTGS2, while ferroptosis inhibition reversed these phenotypes. Our work unveils the tumor promoter LRP2 as a metabolic checkpoint governing ferroptosis in CRLM. Pharmacological disruption of the LRP2-Wnt-GPX4 axis may improve outcomes for metastatic CRC patients.

CRLM and chemotherapy resistance are major drivers of poor clinical outcomes, yet their molecular basis remains incompletely defined [4, 18]. Increasing evidence implicates dysregulated lipid metabolism in CRLM progression [5, 6], highlighting the need for lipid metabolism–related biomarkers and therapeutic targets. Comprehensive insights into CRLM pathogenesis require integration of clinical data, molecular biology, and computational approaches [18]. Public resources such as TCGA and MSigDB have been instrumental in elucidating CRLM biology at a systems level, accelerating the translation of molecular signatures into mechanistic understanding [19–22]. Using an integrated bioinformatics strategy, we identified LRP2, a lipid metabolism–associated gene not previously linked to CRLM, as a functionally relevant driver.

LRP2 belongs to the LDLR family and shares structural similarity with LRP5/6 [11], core components of the Wnt receptor complex. However, its functional relationship with Wnt/β-catenin signaling has not been clarified. Given the established oncogenic role of aberrant Wnt/β-catenin activation in CRC [23–25], we investigated whether LRP2 modulates this pathway. LRP2 silencing markedly attenuated Wnt/β-catenin signaling, as indicated by reduced nuclear β-catenin, decreased GSK-3β phosphorylation, and diminished TOPFlash reporter activity. Untargeted metabolomics further showed that LRP2 depletion profoundly altered glycerophospholipid metabolism, a pathway tightly linked to ferroptosis regulation [26, 27]. Since ferroptosis inhibition is strongly associated with tumor progression and therapy resistance [28–33], we examined whether LRP2-mediated Wnt/β-catenin signaling influences ferroptosis via GPX4 as previously reported in other malignancies [17, 34]. Consistent with this hypothesis, LRP2 knockdown reduced GPX4 expression at both mRNA and protein levels, and Wnt activation restored GPX4 expression in LRP2-deficient cells. Together, these findings define an LRP2–Wnt/β-catenin–GPX4 regulatory axis governing ferroptosis resistance in CRC.

To test whether LRP2-dependent Wnt/β-catenin signaling directly regulates GPX4 transcription, we assessed the impact of LRP2 knockdown on the four core transcription factors of this pathway (TCF/LEF family). LRP2 depletion significantly reduced TCF4, a principal Wnt effector previously shown to bind the GPX4 promoter in other malignancies [17, 35–37]. Unexpectedly, TCF1 expression was also markedly diminished, a change not previously linked to GPX4 regulation. Functional studies revealed that TCF1 overexpression enhanced GPX4 promoter activity in dual-luciferase assays, and mutating the predicted TCF1-binding motif (CTGTGAT) abolished this effect. These results uncover a previously unrecognized role of TCF1 in ferroptosis regulation, suggesting that differential transcription factor usage within the Wnt pathway may underlie heterogeneous ferroptosis responses across CRC subtypes.

While this study provides compelling evidence for the role of LRP2 in CRLM progression and oxaliplatin resistance through the Wnt/β-catenin-GPX4 axis, several limitations merit consideration. First, the precise molecular mechanisms by which LRP2 regulates Wnt/β-catenin activity remain unclear. Future work should determine whether LRP2 interacts directly with Wnt receptors or modulates ligand availability. Second, although we focused on GPX4, LRP2 may also affect additional lipid metabolic pathways and ferroptosis regulators, such as influence on fatty acid uptake, lipid droplet formation and lipase activity. These unresolved questions represent critical directions for subsequent research, which require systematic investigation and may uncover novel targetable vulnerabilities in CRLM.

## Conclusions

**Fig. 8:**
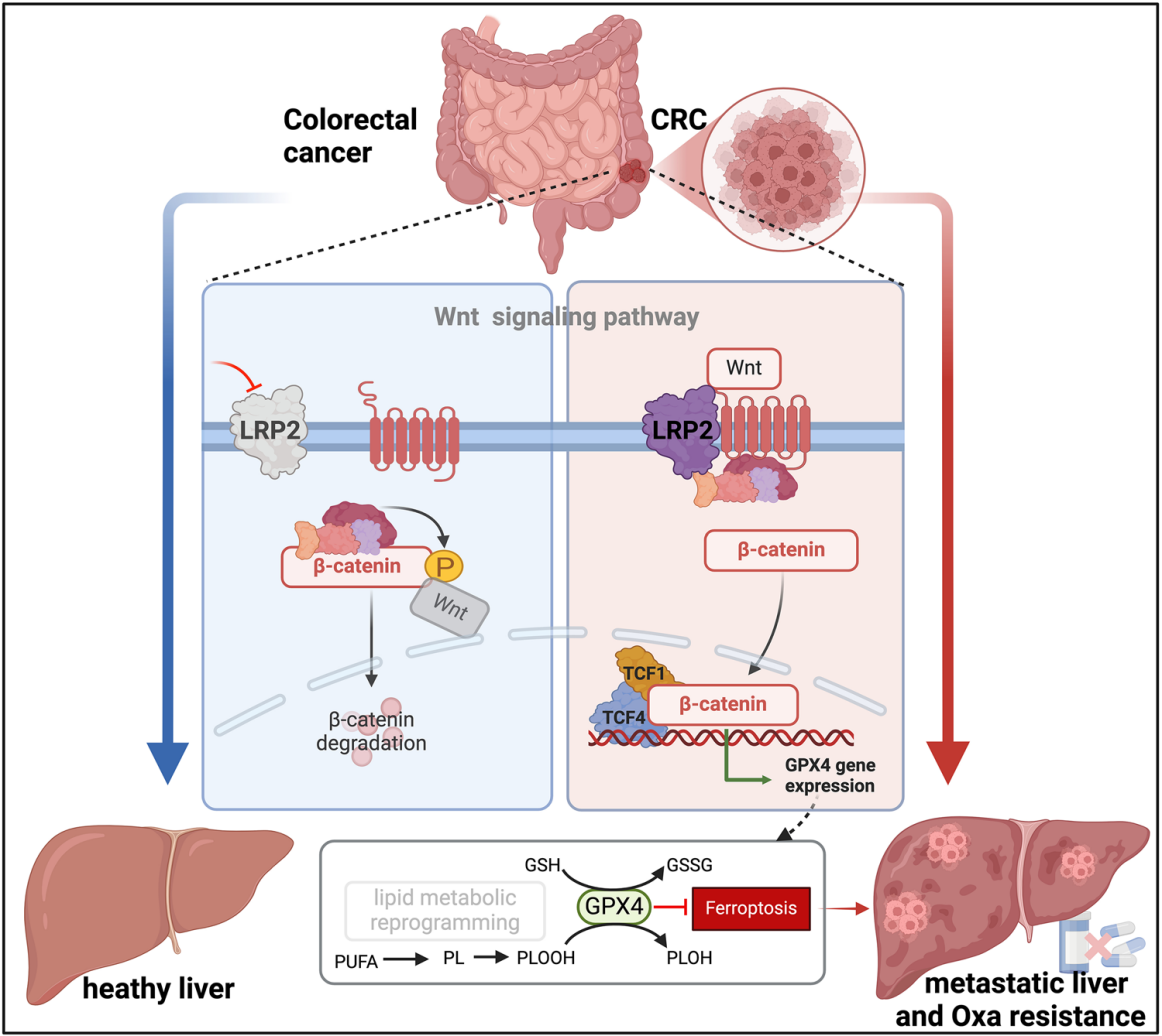
Schematic depiction of the regulation on CRLM progression and chemotherapy sensitivity of oxa by LRP2 in CRC. LRP2 activates the Wnt/β-catenin-TCF1-GPX4 signaling axis to inhibit ferroptosis, thereby promoting CRLM progression and conferring oxaliplatin resistance.

This study identifies LRP2 as a functional driver and prognostic biomarker of CRLM. Mechanistically, LRP2 promotes metastasis and Oxa resistance by activating the Wnt/β-catenin–TCF1–GPX4 axis to suppress ferroptosis (Fig. 8). LRP2 silencing not only restricts tumor progression but also enhances chemosensitivity, suggesting that targeting this pathway may provide new therapeutic opportunities for metastatic CRC.

## Methods

### Bioinformatics analysis

Lipid metabolism–related genes were retrieved from the Molecular Signatures Database (MSigDB, v2023.1). RNA-sequencing data and clinical annotations for CRC were obtained from The Cancer Genome Atlas (TCGA; 39 normal and 401 tumor tissues). Gene symbols were standardized using Perl (v5.32.1). Differential expression analysis was performed with the limma R package (v3.52.4), applying thresholds of |log2FC|≥ 0.5 and false discovery rate (FDR) < 0.05.

Univariate Cox regression identified lipid metabolism–associated genes with prognostic value (*p* < 0.05). Least absolute shrinkage and selection operator (LASSO) Cox regression (glmnet v4.1-6) was used to reduce overfitting. A multivariate Cox model was then constructed, and patient risk scores were calculated as:

$${Risk}\,{score}=\sum \left({\rm{Coefi}}\times {\rm{Expi}}\right)$$

where Coef represents the regression coefficient and Exp the expression level of each gene. Patients were stratified into high- and low-risk groups based on median risk scores. Kaplan–Meier analysis and log-rank tests compared overall survival (OS). Associations between risk scores and clinicopathological features were assessed using chi-square tests (categorical variables) or Wilcoxon rank-sum tests (continuous variables).

Validation was performed using the GEO dataset GSE131418 (878 primary tumors, 257 liver metastases, 15 matched pairs). Probe-level data were mapped to gene-level values and batch effects corrected (sva v3.46.0). LRP2 expression was compared between primary tumors and liver metastases using Mann–Whitney U tests, and in paired samples using Wilcoxon signed-rank tests.

### Human tissues

Tumor and blood samples were obtained from CRC and CRLM patients who underwent surgery at Shanghai Tenth People’s Hospital (2020–2025). All procedures were approved by the hospital ethics committee (Approval No. 22KN87). Tissue arrays included 5 paired CRC and CRLM samples. Plasma LRP2 levels were measured using a commercial ELISA kit (share-bio, Shanghai).

### Cell culture and stable knockdown

Human CRC cell lines SW480 and HCT116 (Cell Bank, Chinese Academy of Sciences, Shanghai) were cultured in DMEM (Gibco, Grand Island, NY, USA) with 10% fetal bovine serum (FBS) at 37 °C in 5% CO₂. Cell identity was verified by Short Tandem Repeat (STR) profiling, and the cells were routinely tested for mycoplasma contamination and confirmed to be negative.

Stable LRP2 knockdown was achieved by lentiviral transduction of short hairpin RNAs (shRNAs) into cells, followed by puromycin (2 μg/mL) selection. The following shRNA sequences were used:

LRP2-KD1: 5′-GCAGCUUACUUGUGACAAUTT-3′

LRP2-KD2: 5′-GCAACUAUGUGGCACACUUTT-3′

A scrambled shRNA served as control. Knockdown efficiency was confirmed by qRT-PCR and western blotting.

### Animal studies

Male BALB/c nude mice (5 weeks old, Beijing Vital River Laboratory) were housed under SPF conditions. All experimental procedures were approved by the Shanghai Tenth People’s Hospital’s Ethics Committee (Approval No. SHDSYY-2024-4709). Mice were randomly assigned to two groups according to body weight to ensure balanced baseline characteristics between groups. For animal studies, investigators were not blinded during the experimental procedures.

For subcutaneous xenografts, HCT116 cells (1.8 × 10^6^ cells in 100 μL PBS) were injected into the right flank (6 mice per group). Tumor growth was measured starting from day 4 post-inoculation. Oxaliplatin (7.5 mg/kg, intraperitoneally) was administered every three days from day 6 for totally three doses. Tumor volume was calculated as: Tumor Volume = 1/2 × Length × Width^2^. Mice were humanely euthanized on day 15, and tumors were excised, weighed, and processed for further analysis.

For liver metastasis models, HCT116 cells (1 × 10^6^ cells in 50 μL PBS) were injected into the spleen of anesthetized nude mice (5 mice per group), followed by splenectomy after maintained in a supine position for 5 min to facilitate cell dissemination. Livers were harvested on day 21 for macroscopic and histological analysis.

### Cell proliferation viability assays

Cell proliferation was assessed using CCK-8 assays (B34304, Bimake) at five time points (0, 24, 48, 72, 96, 120 and 144 h) and EdU incorporation (YF®555 Click-iT Kit, US Everbright). Viability was determined after exposure to oxaliplatin (1.875–30 μM) for 48 h using CCK-8.

### Colony formation assay

CRC cells (1000/well) were seeded in 12-well plates and cultured for 14 days. Colonies (>50 cells) were fixed, stained with crystal violet, and counted using Image J 1.54.

### Oxidative stress and ferroptosis assays

The GSH/GSSG ratio was measured using a commercial kit (S0053, Beyotime), which strictly following the manufacturer’s protocol.

For MDA levels measurement, Lipid Peroxidation Assay Kit (S0131S, Beyotime) was used according to the manufacturer’s instructions.

To assess cellular lipid peroxidation, CRC cells were analyzed by C11-BODIPY 581/591(Invitrogen) staining and flow cytometry (Becton Dickinson).

Cell death was quantified by propidium iodide (1:1000; Invitrogen) staining for 5 min at room temperature followed by flow cytometry.

For ultrastructure assessment, cells were fixed in situ with 2.5% glutaraldehyde in 0.1 M phosphate buffer (pH 7.4) for 30 min at room temperature. Resin embedding and section preparation (70 nm) were then prepared, and assessed by transmission electron microscopy (HITACHI, Tokyo, Japan) at 80 kV.

### Migration assays

Wound-healing assays were performed in confluent monolayers scratched with pipette tips. Following 0, 24 and 48 h after scratched, wound closure was quantitatively assessed. The percentage of wound closure at each time point (T) was calculated using the formula: % Wound Closure = [(Area₀h − Area_T)/Area₀h] × 100%.

Transwell migration assays used a double-chamber culture apparatus with an 8-μm pore microporous membrane. Briefly, 5 × 10⁴ cells suspended in serum-free medium were seeded in the upper chamber, while the lower chamber contained complete medium supplemented with 20% fetal bovine serum (FBS; Gibco) as a chemoattractant. After 48 h incubation, cells remaining on the upper surface of the membrane were removed with cotton swabs. Cells that had migrated to the lower surface were fixed in 4% paraformaldehyde for 15 min and stained with 0.1% crystal violet (Sigma-Aldrich) for 30 min at room temperature.

### Protein extraction and western blotting

Proteins were extracted with RIPA buffer and quantified using a BCA Protein Assay Kit (Beyotime, Shanghai). Equal amounts of protein (20 μg per lane) were separated by SDS-PAGE (10% gels) and subsequently transferred to PVDF membranes (Millipore, USA). The membranes were probed with primary antibodies diluted in blocking buffer overnight at 4 °C after blocked with 5% non-fat milk in Tris-buffered saline containing 0.1% Tween-20 (TBST) for 2 h at room temperature. After three washes with TBST (10 min each), the membranes were incubated with horseradish peroxidase (HRP)-conjugated secondary antibodies for 1 h at room temperature. Protein bands were visualized using an enhanced chemiluminescence (ECL) detection reagent (Millipore, USA) and imaged with a Tanon 5200 Luminescent Imaging System (Tanon, China).

The primary antibodies used in this study were as follows: anti-LRP2 (Abmart, # PK10185), anti-β-actin (Abmart, # T40104), anti-β-catenin (Epitomics, # 1247-1), anti-Histone H4 (Abcam, # ab31830), anti-pGSK-3β (CST, # 9323), anti-GSK-3β (Abmart, # TA5016), anti-GPX4(Abmart, # T56959), anti-ACSL4(Abmart, # TD12141), anti-SLC7A11(Abmart, # T57046), anti-TCF1(CST, # 2203), anti-TCF4(CST, # 2569).

### Quantitative PCR

Total RNA was extracted with RNA-Quick Purification Kit (YiShan Biotechnology, Shanghai, China) and reverse transcribed using PrimeScript RT Master Mix (Takara, Japan). Quantitative PCR was performed using SYBR Green Mix (Takara, Japan). The mRNA levels of target genes were normalized to GAPDH mRNA. The primer sequences are listed as follow:

LRP2 primers

Forward 5’-TCATGTGGCAATGGAGAGTG-3’

Reverse 5’-ACTGGTAACCACCGCAGGTC-3’

GAPDH primers

Forward 5’-TGCACCACCAACTGCTTAGC-3’

Reverse 5’-GGCATGGACTGTGGTCATGAG-3’

GPX4 primers

Forward: 5′-TGGGAAATGCCATCAAGTGG-3′

Reverse: 5′-GGTCCTTCTCTATCACCAGGGG-3′

### Immunohistochemistry (IHC)

Formalin-fixed, paraffin-embedded tissues were sectioned at 3 μm thickness, deparaffinized in xylene, and rehydrated through a graded ethanol series. Antigen retrieval was performed using sodium citrate buffer (10 mM, pH 6.0) followed by endogenous peroxidase blocking with 3% H₂O₂ for 15 min. After PBS washes, sections were blocked with 5% BSA for 2 h at room temperature and incubated with antibodies against LRP2, Ki-67, and PTGS2 (Abmart, China) overnight at 4 °C, followed by HRP-conjugated secondary antibodies and DAB detection (Sigma, USA). Nuclei were counterstained with hematoxylin. Images were acquired using a Leica microscope (Japan).

### Metabolomics

Untargeted metabolomics was performed using UHPLC-Q Exactive Orbitrap (Metabo-Profile, Shanghai). Processed metabolites were analyzed for differential abundance and pathway enrichment.

### Dual-luciferase assays

TOPFlash/FOPFlash and GPX4 promoter constructs (wild-type and mutants) were transfected with or without TCF1 expression plasmids. Luciferase activity was measured using a Dual-Luciferase Reporter Assay Kit (RG029S, Beyotime).

### Statistics analysis

The sample sizes were selected based on previous literature, and common practice in the field. All experiments were performed with at least three independent biological replicates for in vitro studies and at least five animals per group for in vivo studies.

Data are expressed as mean ± SD from ≥3 independent experiments unless otherwise stated. Comparisons between two groups were performed using Student’s *t* test. For comparisons involving more than two groups, one-way or two-way ANOVA was used to evaluate overall differences, followed by Tukey’s multiple comparison test. A *P* value < 0.05 was considered significant. All statistical computations were performed with GraphPad Prism version 9.0 (GraphPad Software, San Diego, CA).

## Supplementary information

Supplementary Figure 1

Supplementary Figure 2

Supplementary Figure 3

Supplementary Figure 4

Supplementary Figure legends

Full-length uncropped Western blots

## Data Availability

Data generated in this study can be obtained from the corresponding author upon reasonable request.

## Code availability

The custom R scripts used for data analysis and figure generation in this study are available from the corresponding author upon reasonable request.
