# Supplementary figures and images for: LRP2-mediated regulation of ferroptosis through the Wnt/β-catenin–GPX4 axis in colorectal cancer liver metastasis and chemoresistance
Source: Cell Death Discov. 2026 May 29;12:320. doi: 10.1038/s41420-026-03161-4 (PMC13424328; doi:10.1038/s41420-026-03161-4)

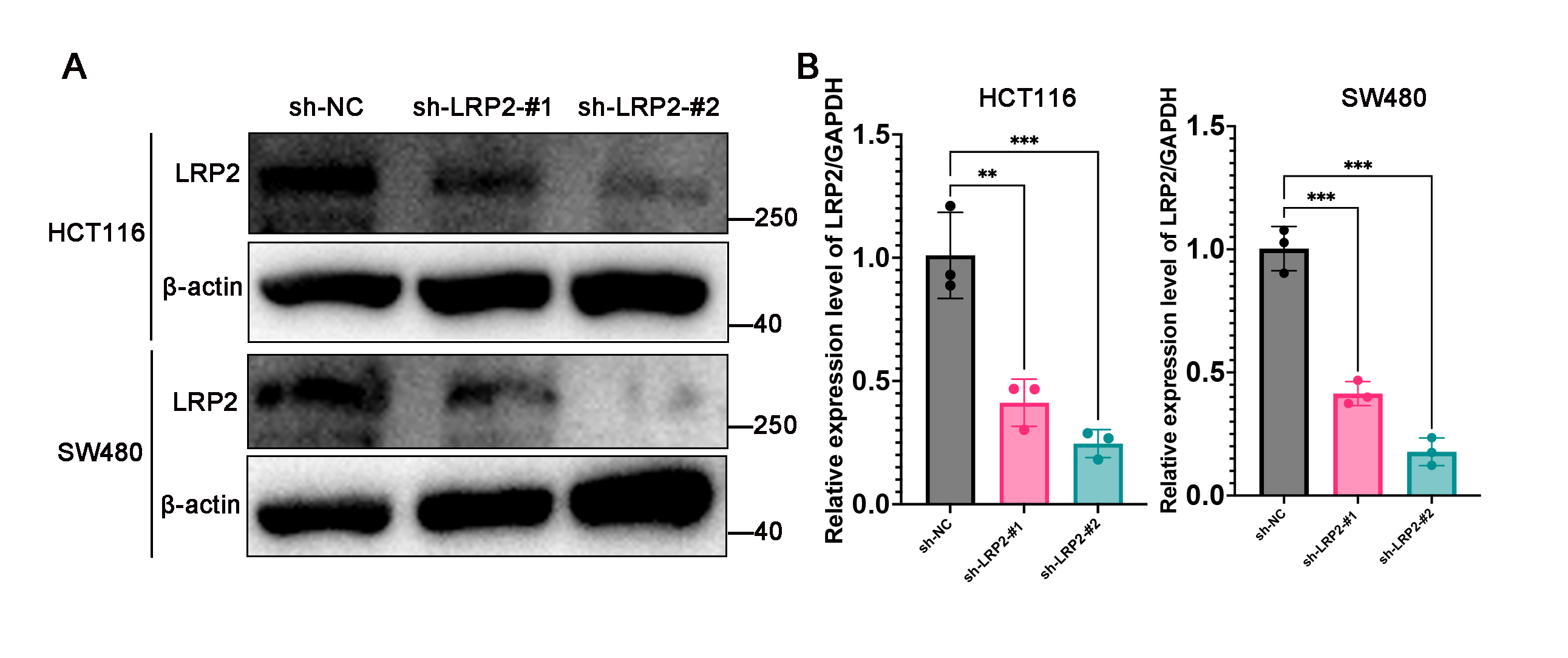

Supplement: Supplementary file 2 — Supplementary Figure 2 [file 41420_2026_3161_MOESM2_ESM.tif]

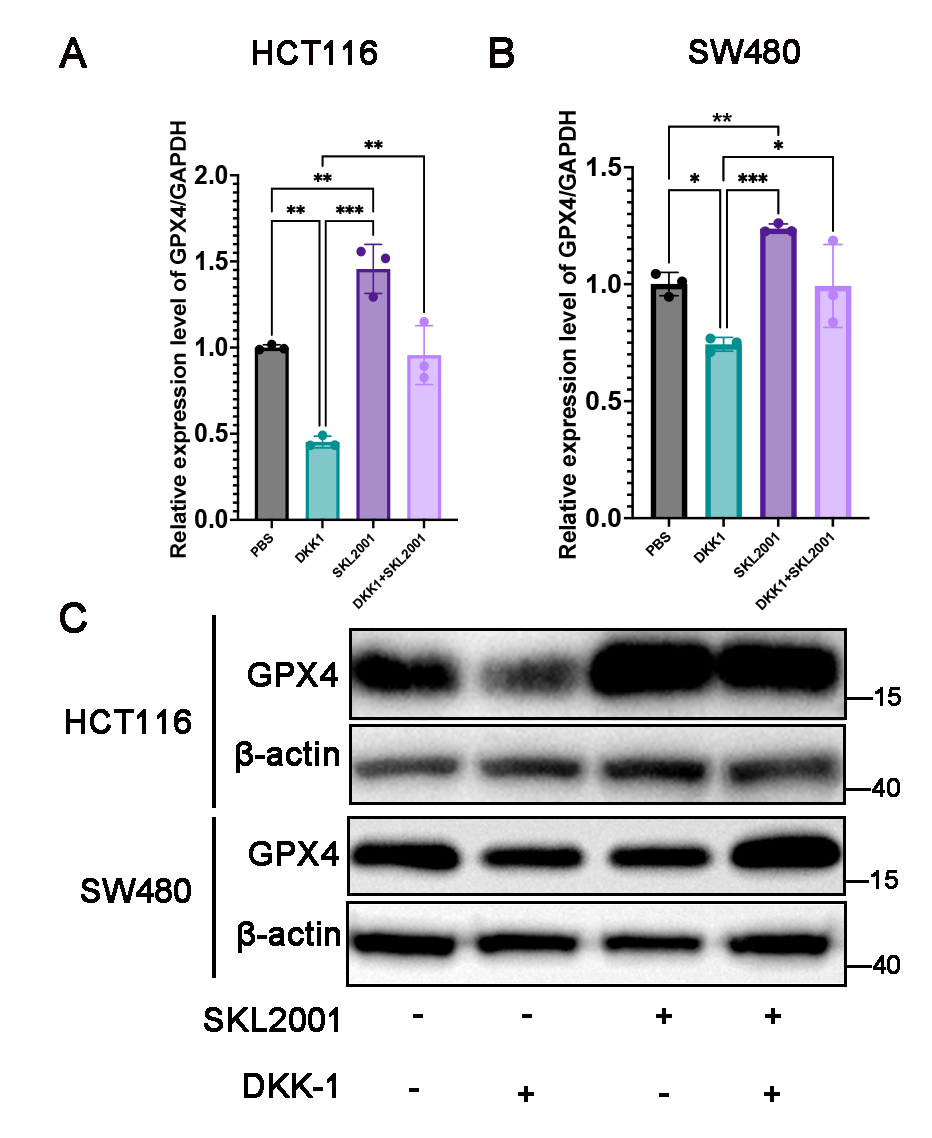

Supplement: Supplementary file 3 — Supplementary Figure 3 [file 41420_2026_3161_MOESM3_ESM.tif]

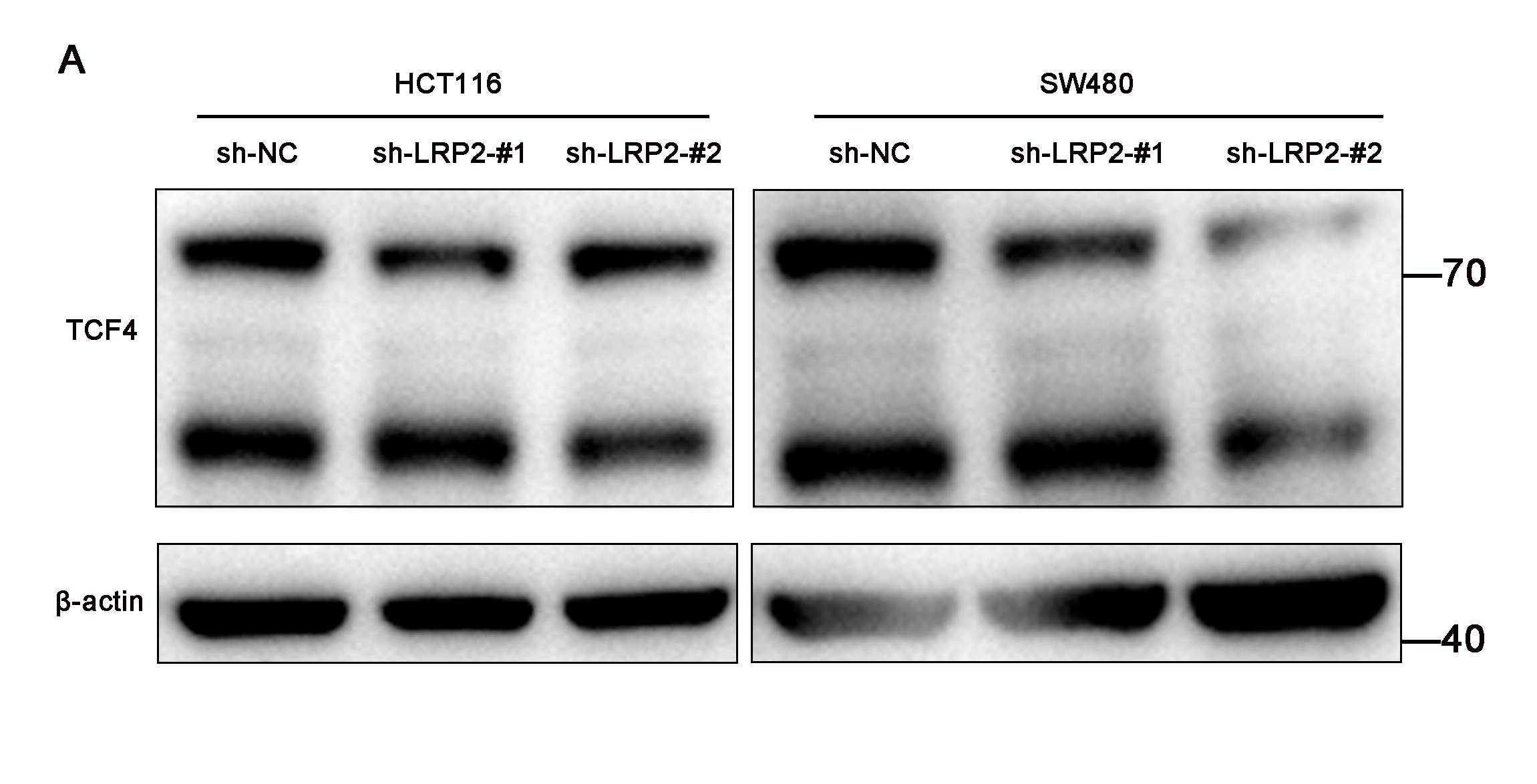

Supplement: Supplementary file 4 — Supplementary Figure 4 [file 41420_2026_3161_MOESM4_ESM.tif]
