# Supplementary material for: LRP2-mediated regulation of ferroptosis through the Wnt/β-catenin–GPX4 axis in colorectal cancer liver metastasis and chemoresistance
Source: Cell Death Discov. 2026 May 29;12:320. doi: 10.1038/s41420-026-03161-4 (PMC13424328; doi:10.1038/s41420-026-03161-4)
Supplement: Supplementary file 5 — Supplementary Figure legends [file 41420_2026_3161_MOESM5_ESM.docx]

**Supplementary Figure 1. Expression of other prognostic genes.** (A-F) AGMO expression among patients grouped based on distinct clinical features. (G-L) ENO3 expression among patients grouped based on distinct clinical features. (M-R) FABP4 expression among patients grouped based on distinct clinical features. (S-X) TERT expression among patients grouped based on distinct clinical features.

**Supplementary Figure 2. LRP2 knockdown efficiency in CRC cells.** (A) Representative Western blot and (B) qRT-PCR analysis of LRP2 expression in sh-NC and sh-LRP2 CRC cells. Data represent mean ± SD of three independent experiments (n=3, **P < 0.01, ***P < 0.001, Student's t-test).

**Supplementary Figure 3. Wnt signaling positive regulates GPX4 expression in CRC cells.** (A, B) qRT-PCR and (C) western blot analysis of GPX4 expression in WT CRC cells with or without SKL2001 or DKK-1. Data represent mean ± SD of three independent experiments (n=3, *P < 0.05, **P < 0.01, ***P < 0.001, Student's t-test).

**Supplementary Figure 4. TCF4 expression regulated by the LRP2 in CRC cells.** (A) Representative western blot analysis of TCF4 expression in sh-NC and sh-LRP2 CRC cells.
