# Supplementary material for: LRP2-mediated regulation of ferroptosis through the Wnt/β-catenin–GPX4 axis in colorectal cancer liver metastasis and chemoresistance
Source: Cell Death Discov. 2026 May 29;12:320. doi: 10.1038/s41420-026-03161-4 (PMC13424328; doi:10.1038/s41420-026-03161-4)

# Supplementary materials

Supplementary materials. Full-length uncropped Western blots.
The red box indicates the cropped area shown in the main figure.

Corresponding to Figure 2F (LRP2).

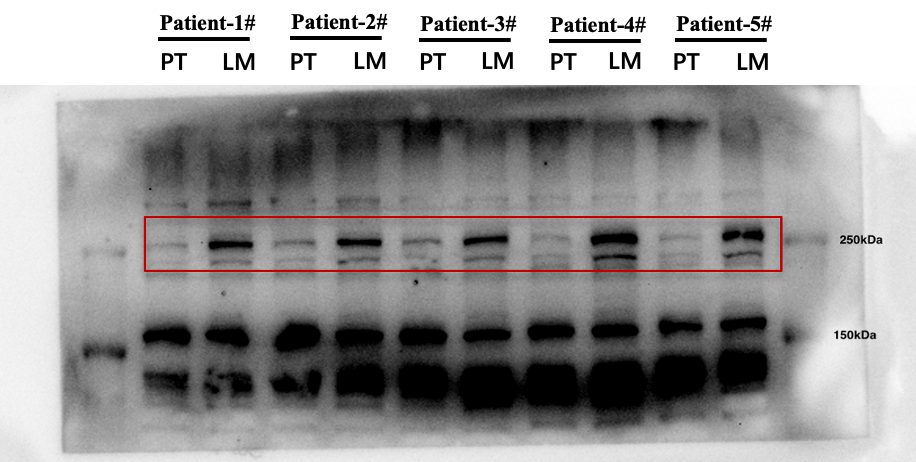


Corresponding to Figure 2F (β-actin).

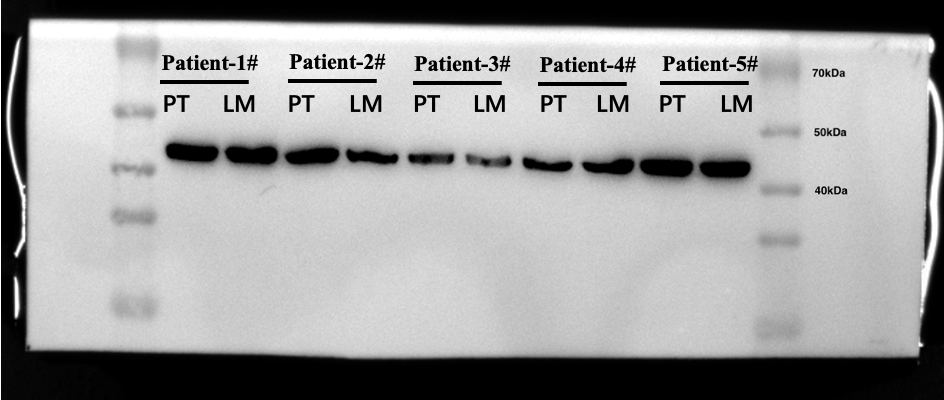


Corresponding to Figure 7C (HCT116, β-catenin).

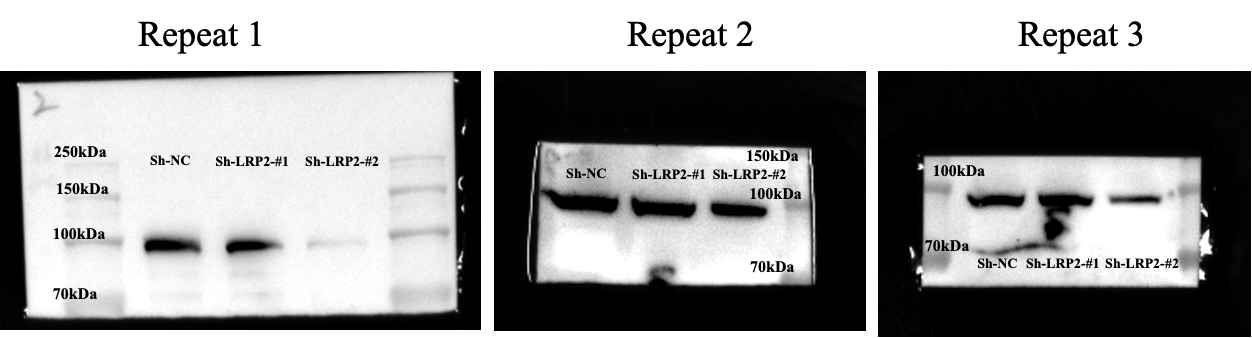


Corresponding to Figure 7C (HCT116, Histone H4).

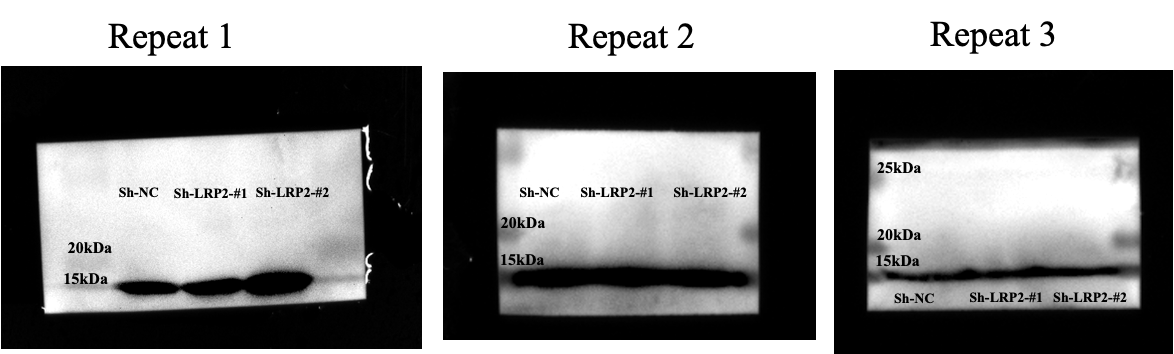


Corresponding to Figure 7C (SW480, β-catenin).

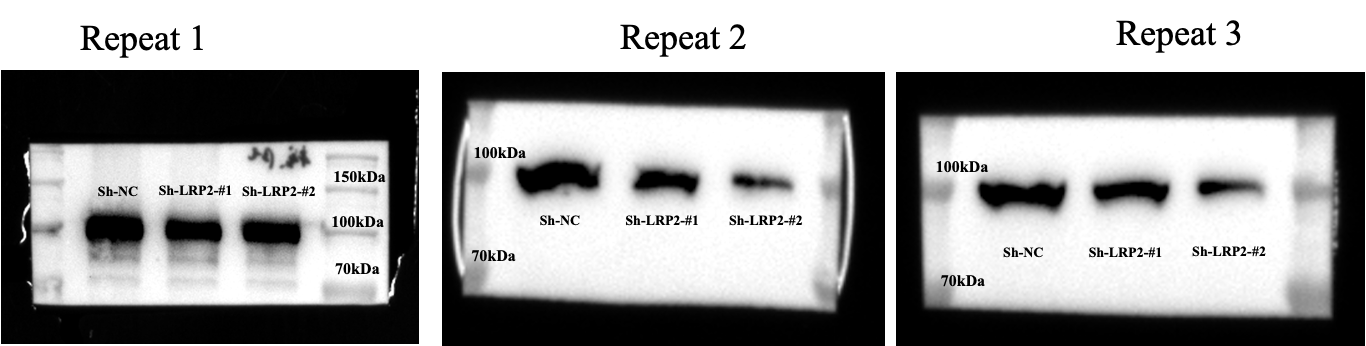


Corresponding to Figure 7C (SW480, Histone H4).

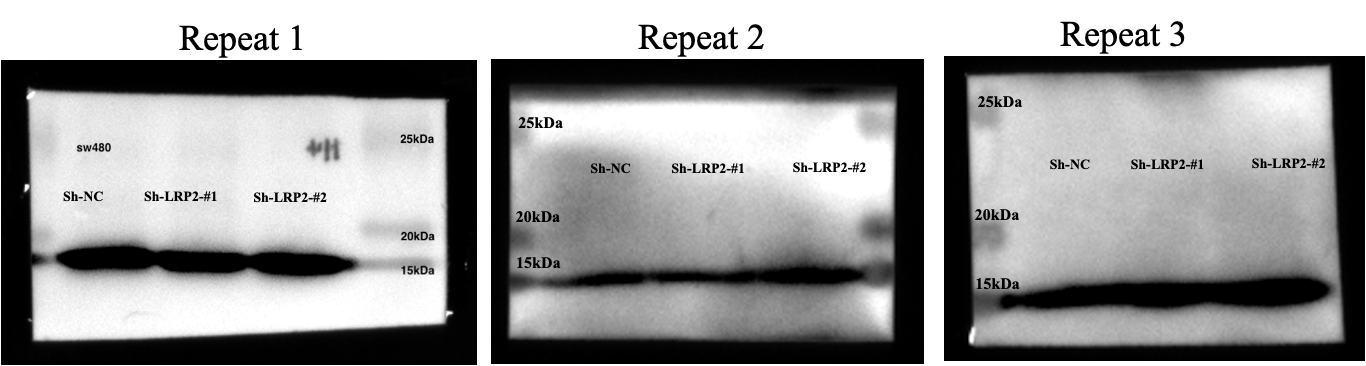


Corresponding to Figure 7D (HCT116, β-catenin).

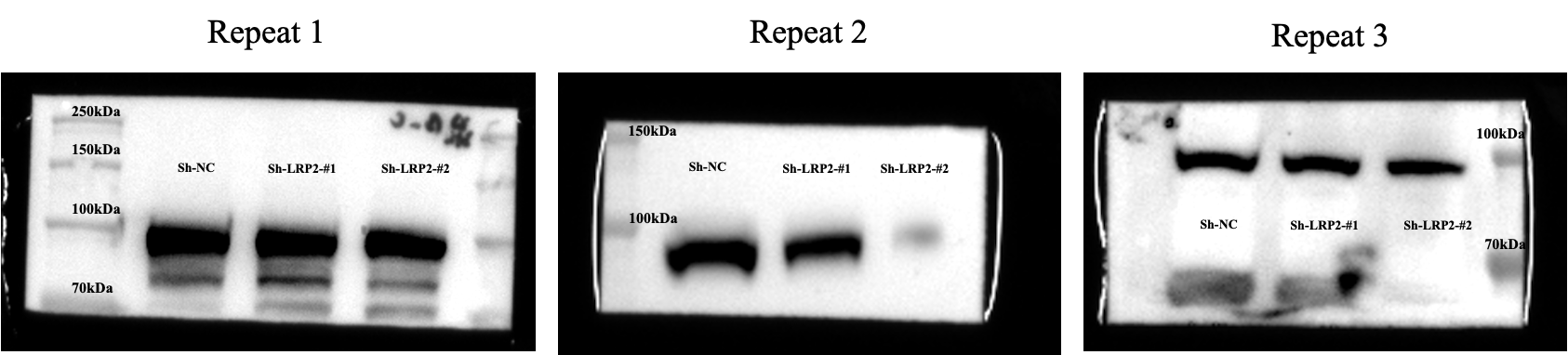


Corresponding to Figure 7D (HCT116, β-actin).

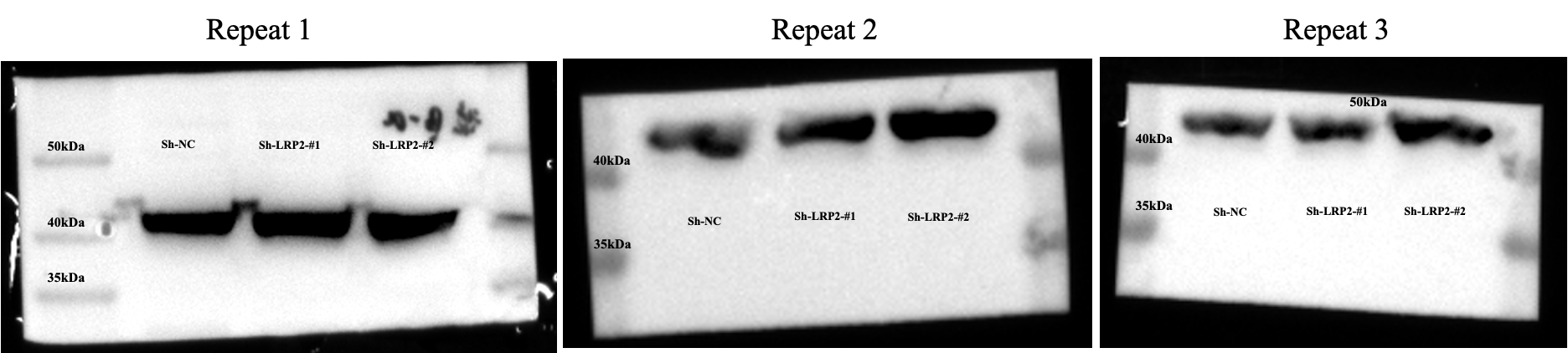


Corresponding to Figure 7D (sw480, β-catenin).

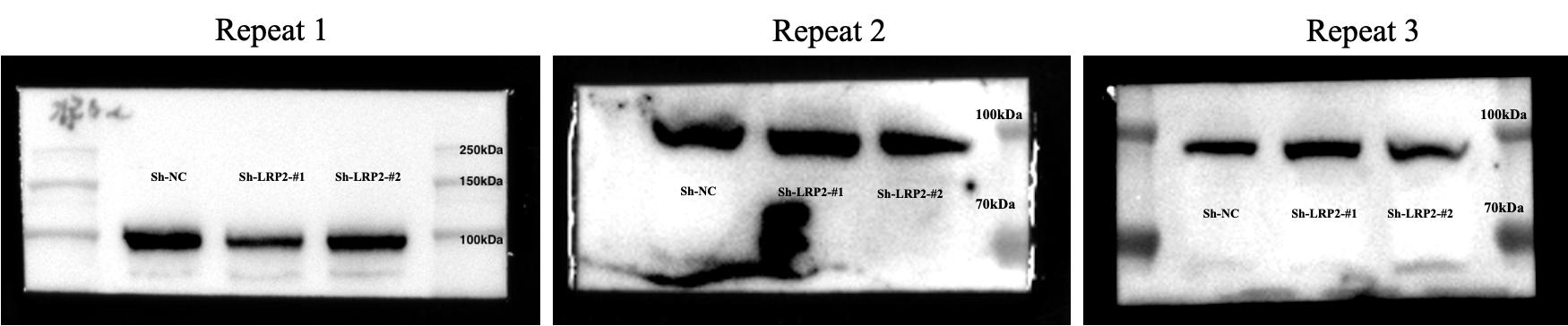


Corresponding to Figure 7D (sw480, β-actin).

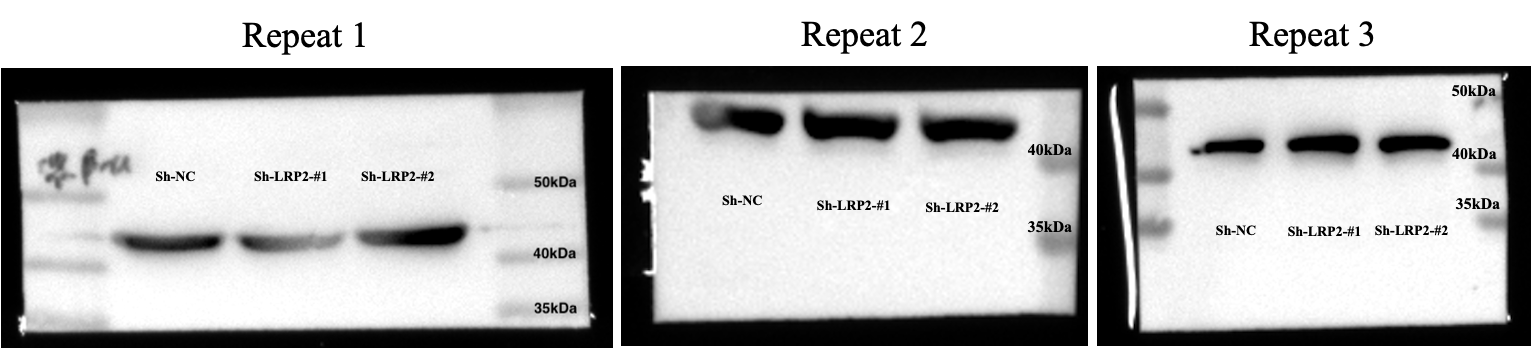


Corresponding to Figure 7E (HCT116, β-catenin).

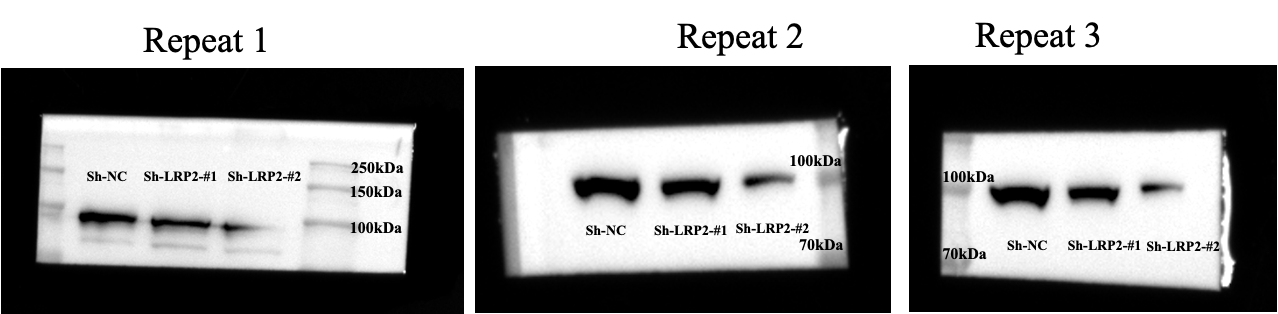


Corresponding to Figure 7E (HCT116, pGSK-3β).

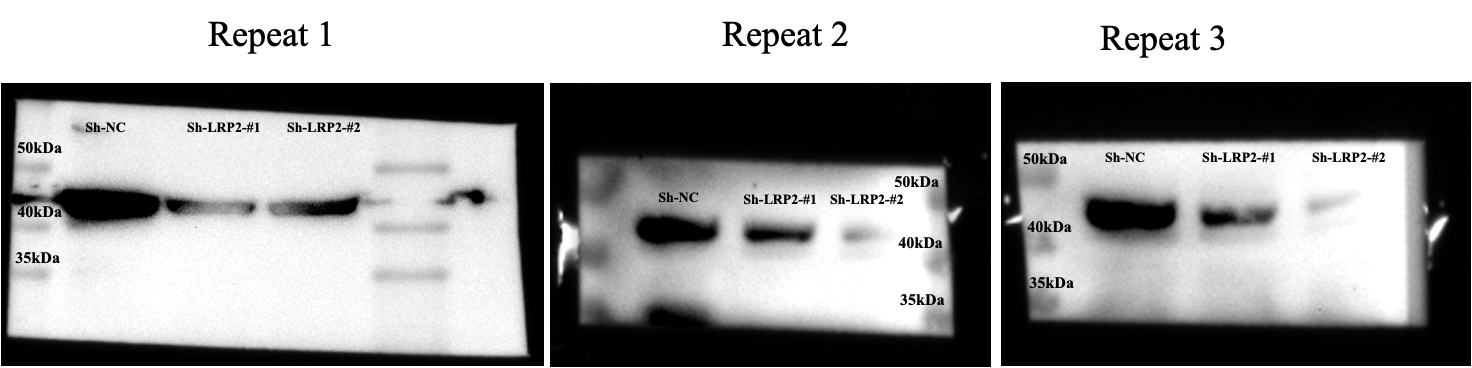


Corresponding to Figure 7E (HCT116, GSK-3β).

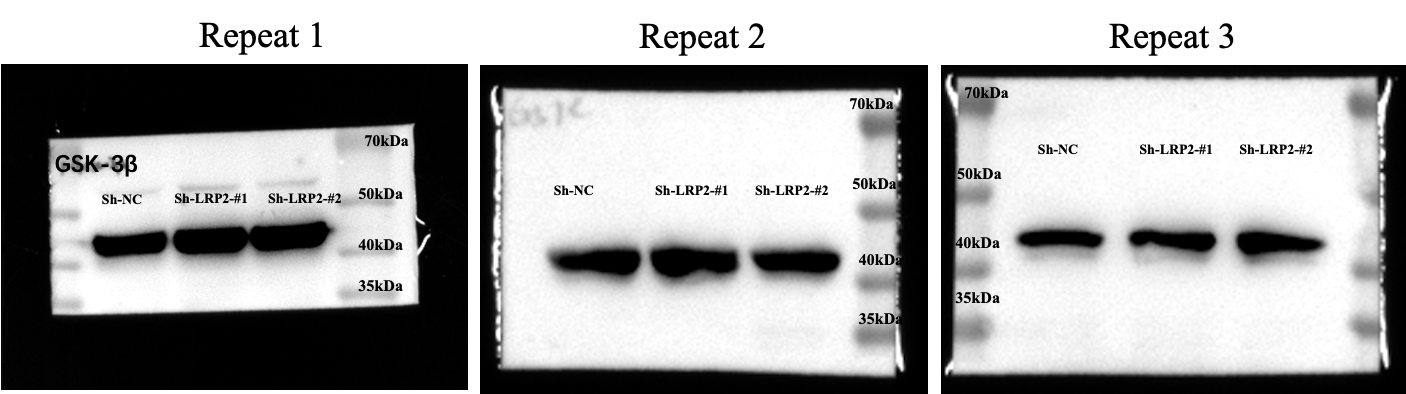


Corresponding to Figure 7E (HCT116, GPX4).

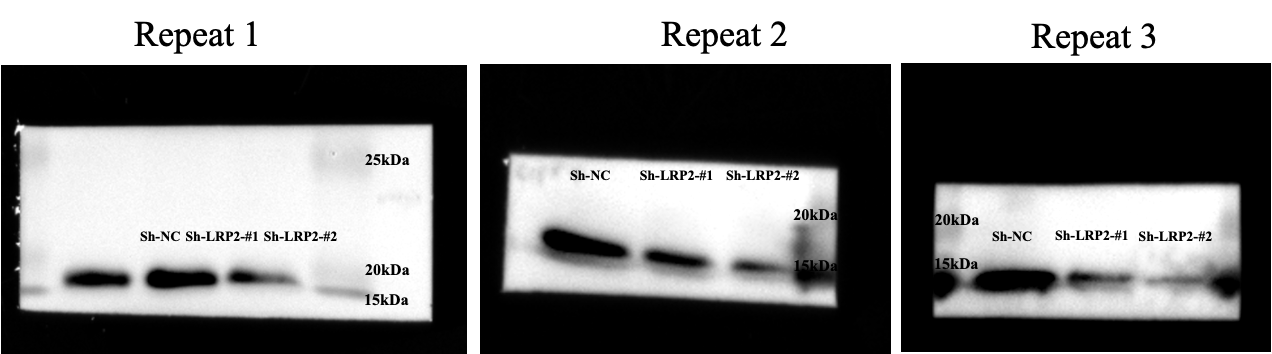


Corresponding to Figure 7E (HCT116, ACSL4).

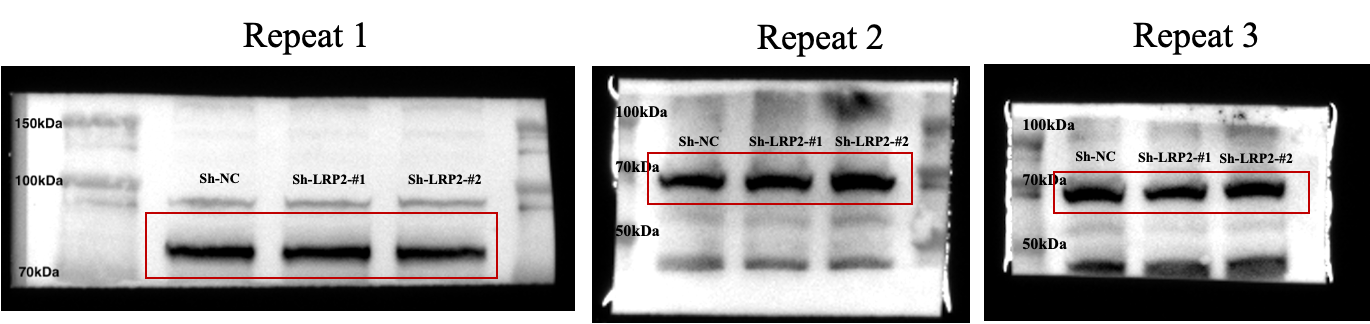


Corresponding to Figure 7E (HCT116, SLC7A11).

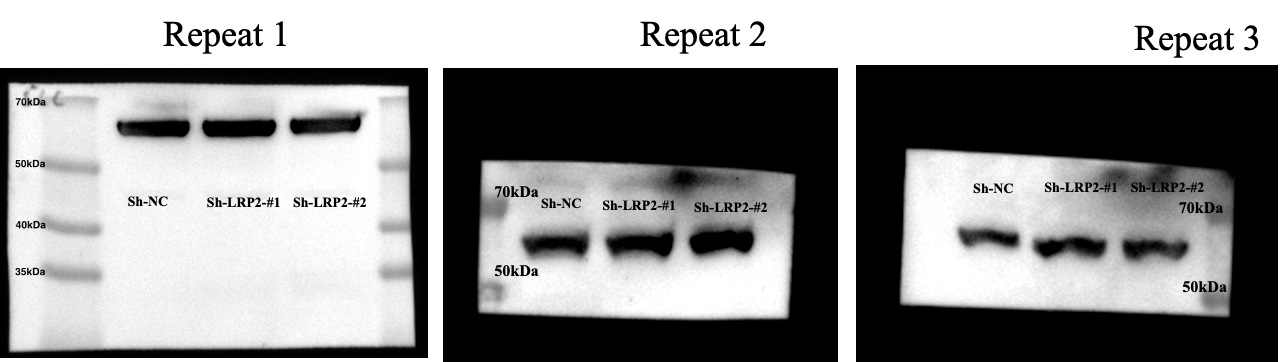


Corresponding to Figure 7E (HCT116, β-actin).

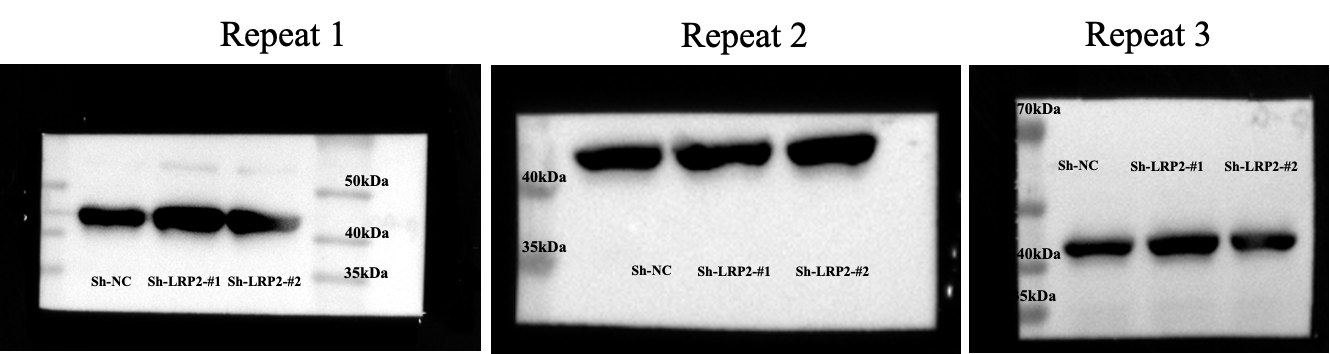


Corresponding to Figure 7E (SW480, β-catenin).

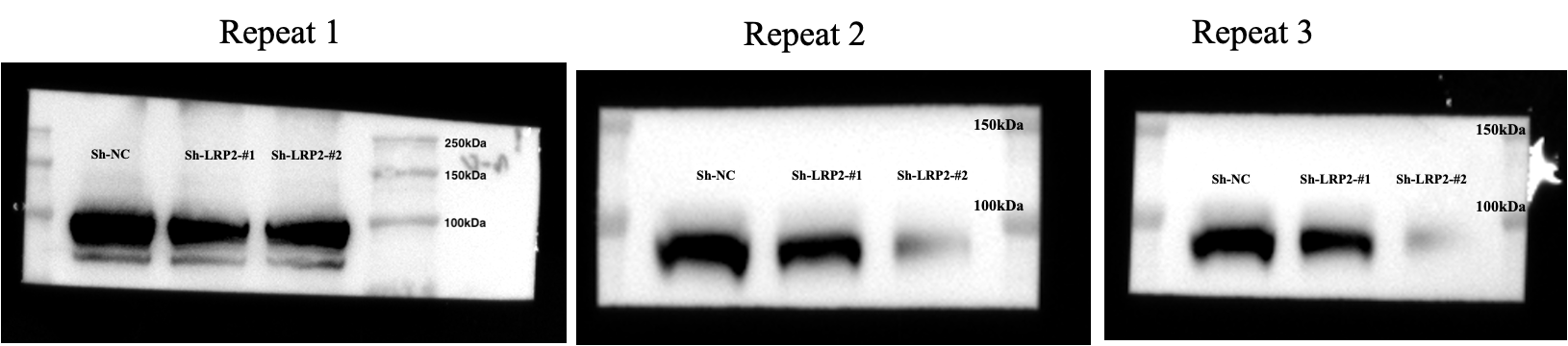


Corresponding to Figure 7E (SW480, pGSK-3β).

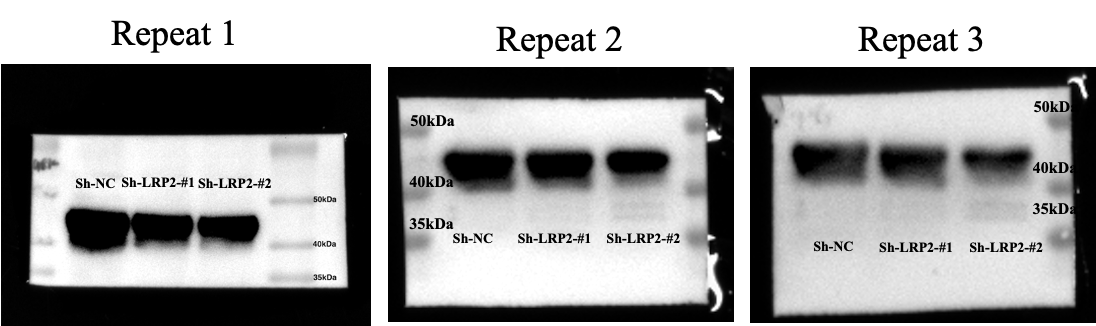


Corresponding to Figure 7E (SW480, GSK-3β).

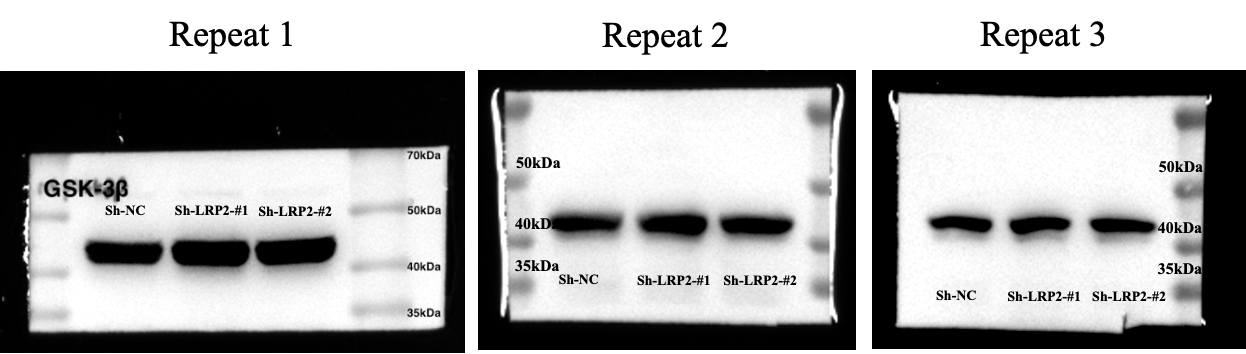


Corresponding to Figure 7E (SW480, GPX4).

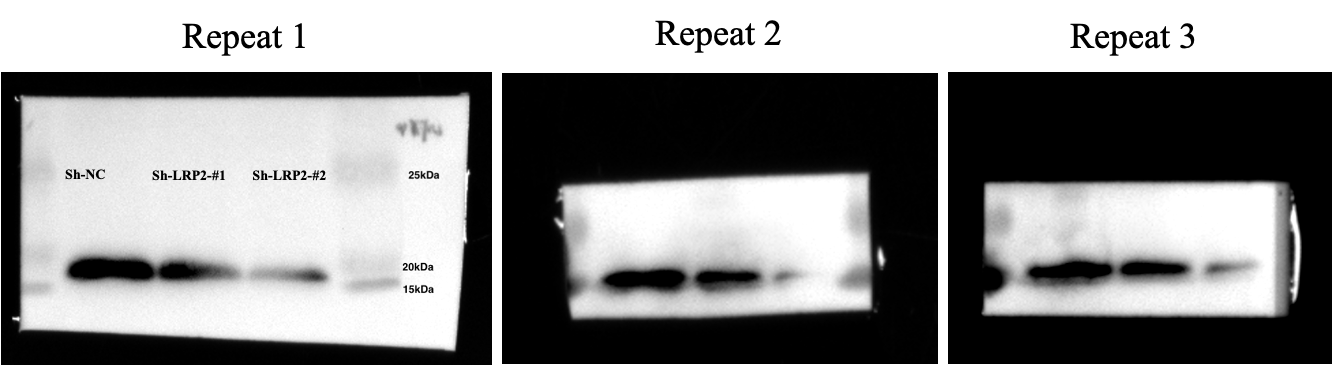


Corresponding to Figure 7E (SW480, ACSL4).

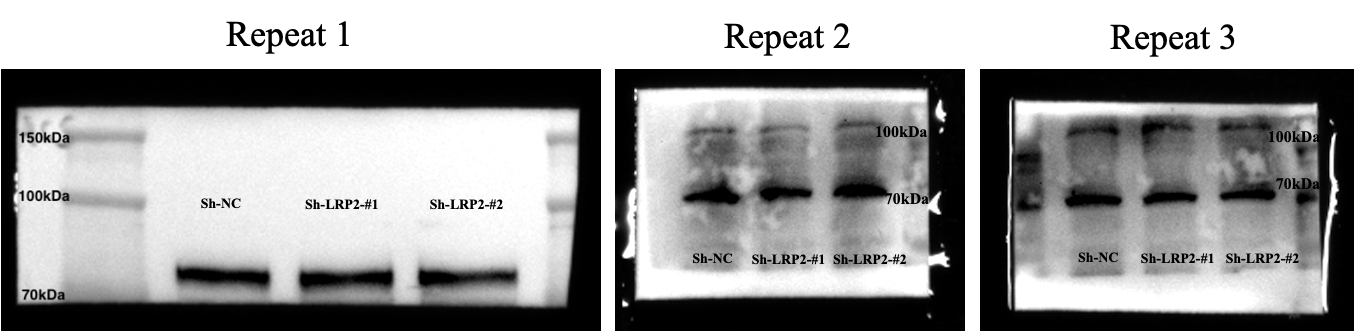


Corresponding to Figure 7E (SW480, SLC7A11).

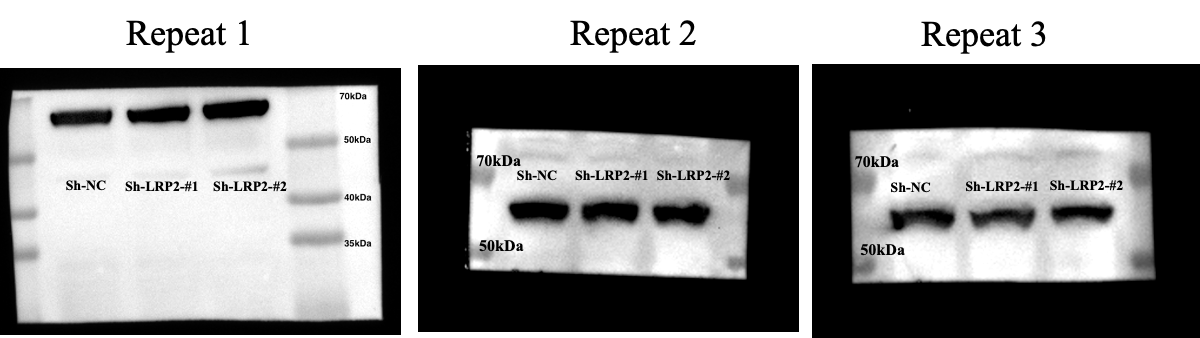


Corresponding to Figure 7E (SW480, β-actin).

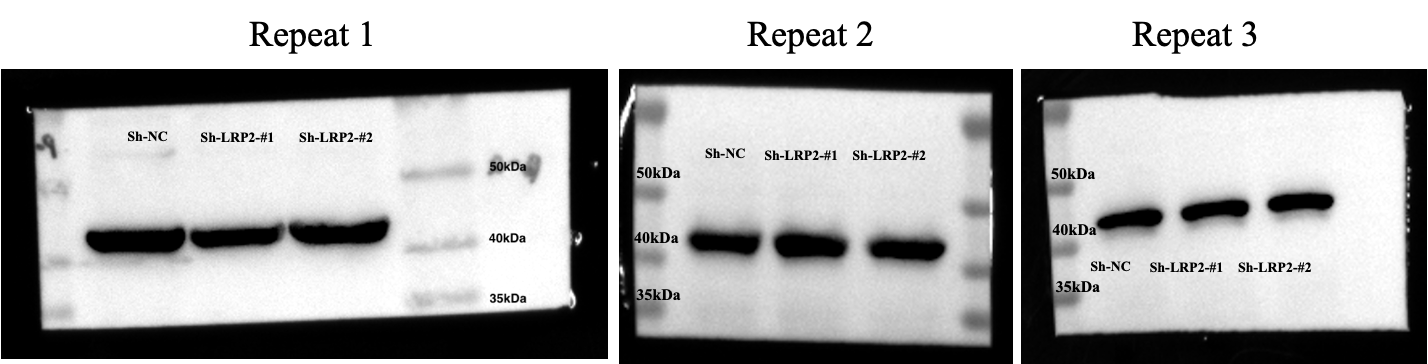


Corresponding to Figure 7F (HCT116, GPX4).

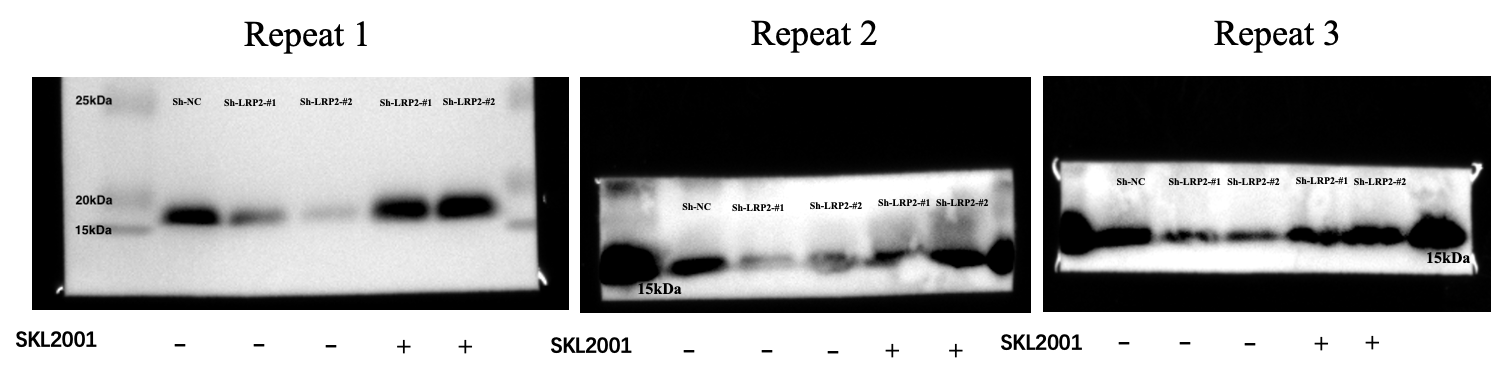


Corresponding to Figure 7F (HCT116, β-actin).

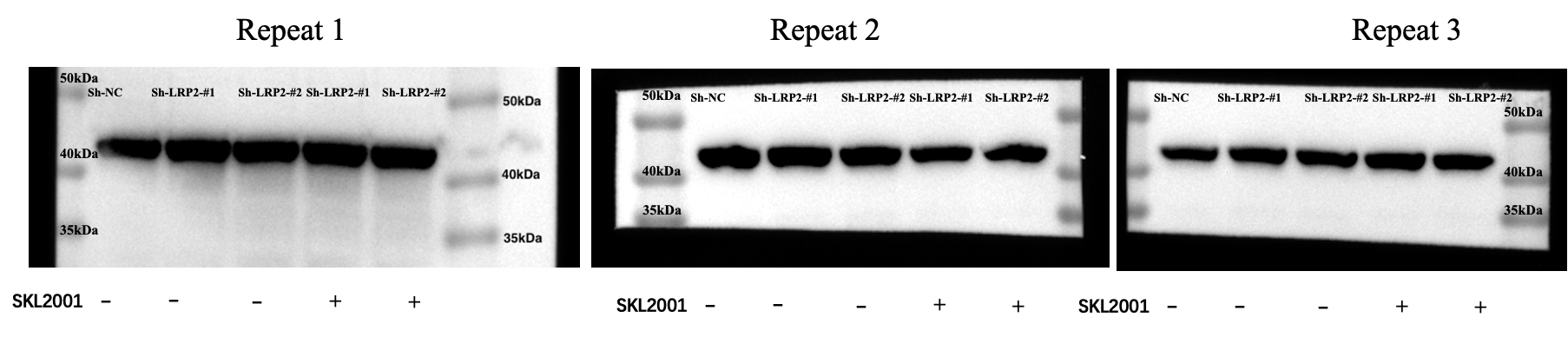


Corresponding to Figure 7F (SW480, GPX4).

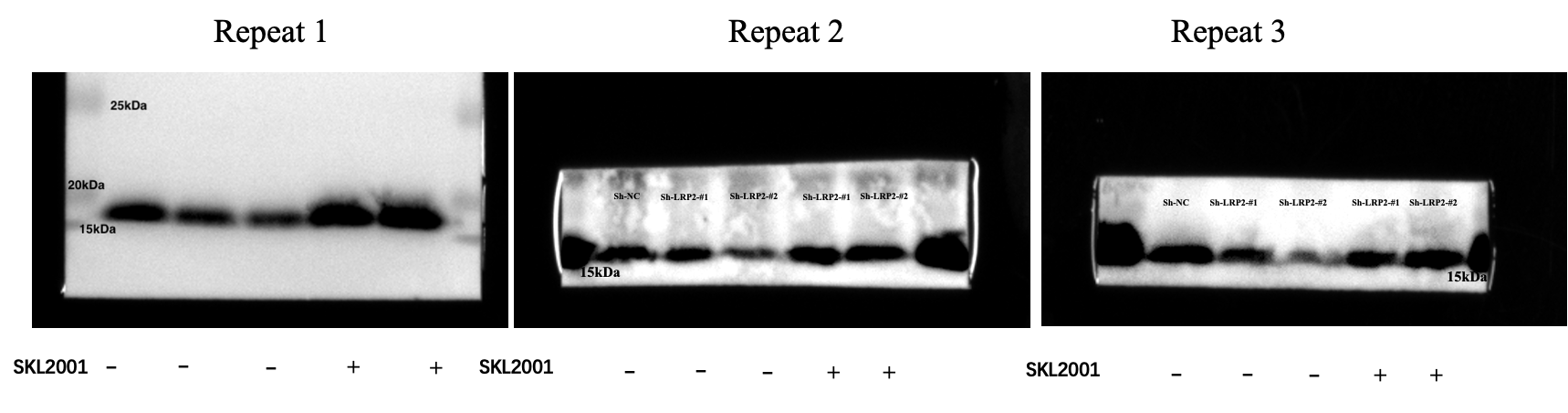


Corresponding to Figure 7F (SW480, β-actin).

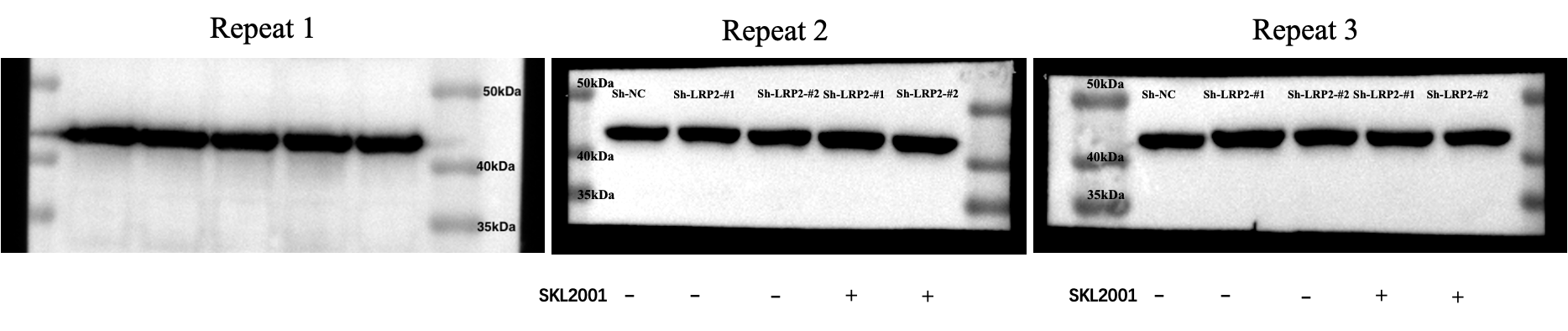


Corresponding to Figure 7K (HCT116, TCF1).

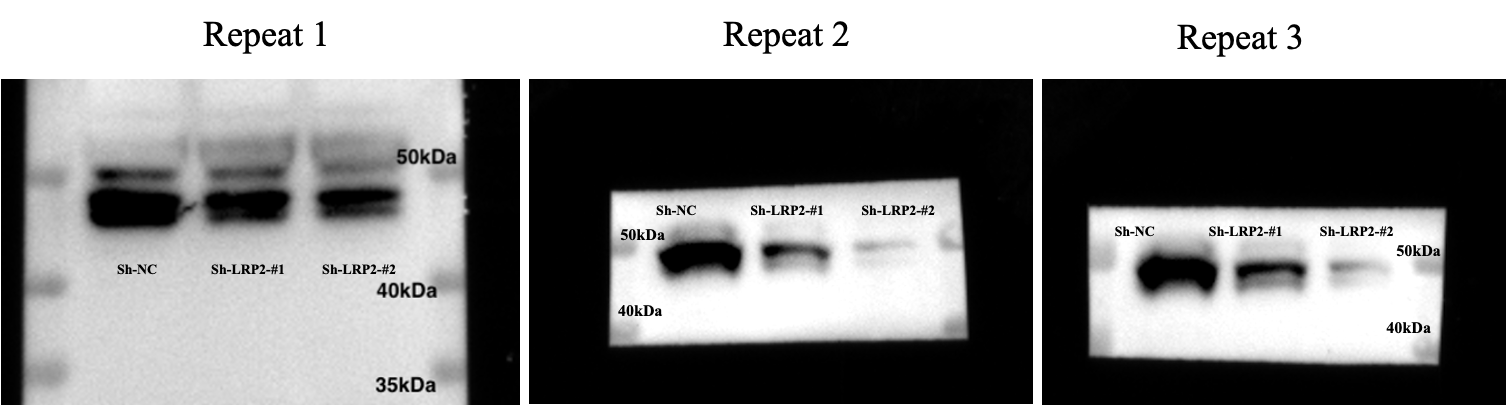


Corresponding to Figure 7K and Supplementary Figure 4A (HCT116β-actin).

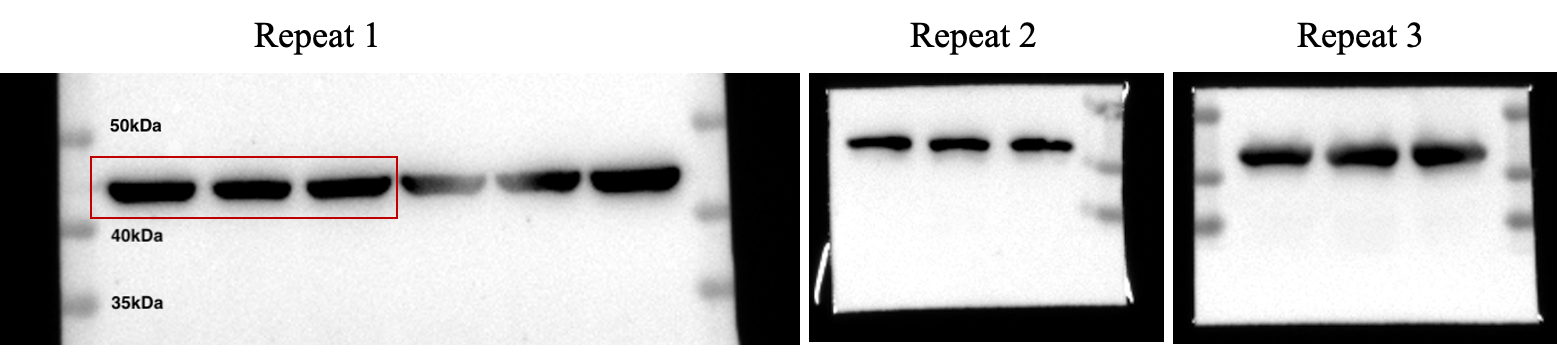


Corresponding to Figure 7K (SW480, TCF1).

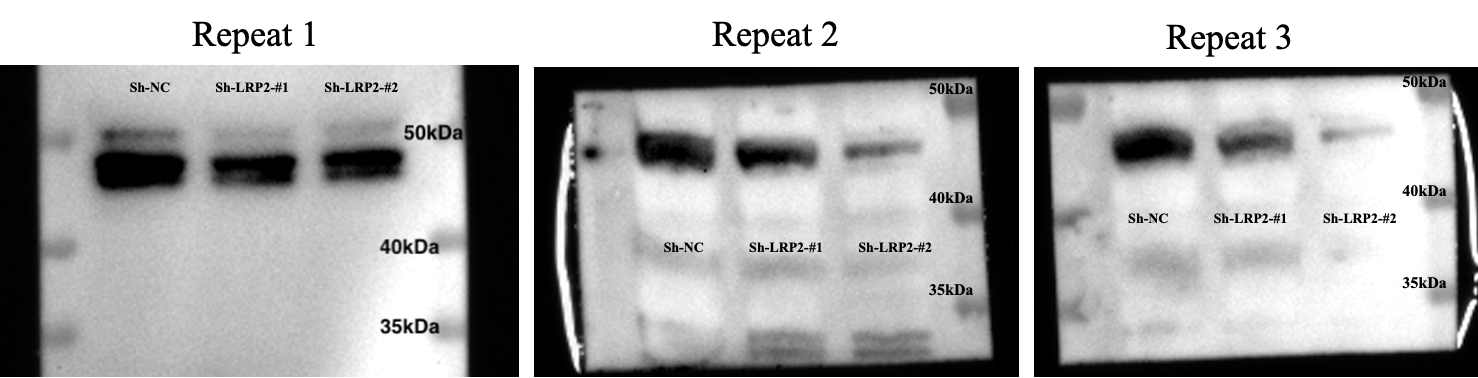


Corresponding to Figure 7K and Supplementary Figure 4A (sw480β-actin).

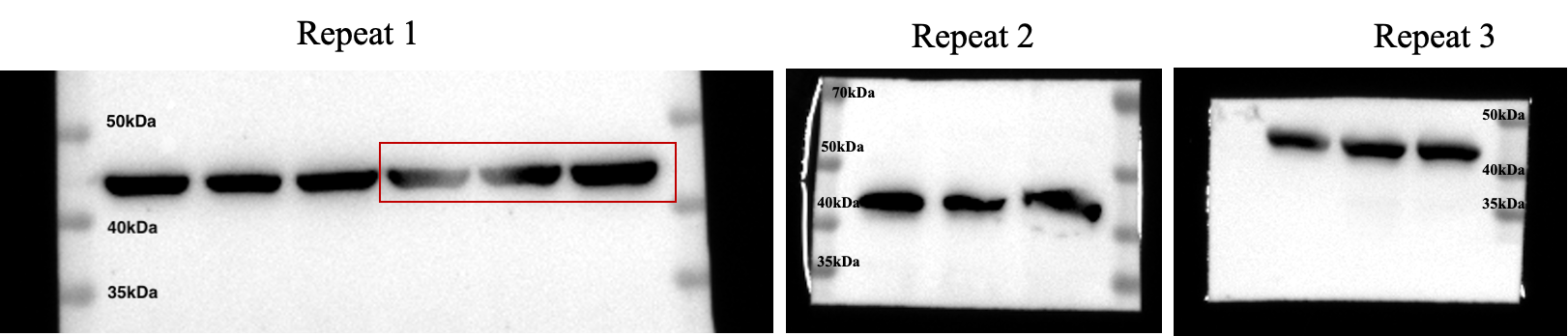


Corresponding to Figure 7L (HCT116 GPX4).

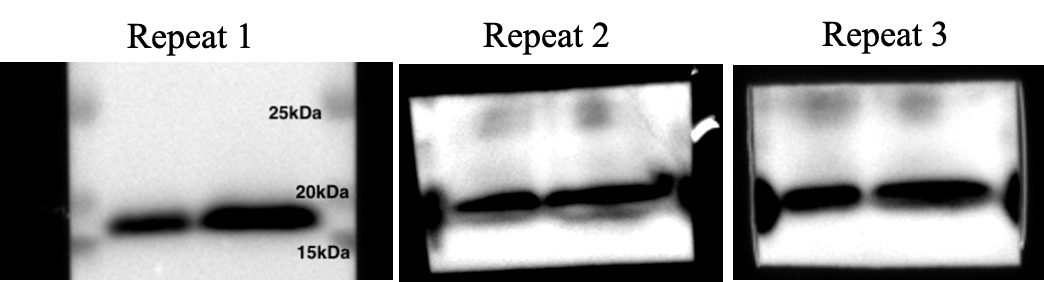


Corresponding to Figure 7L (HCT116 TCF1).

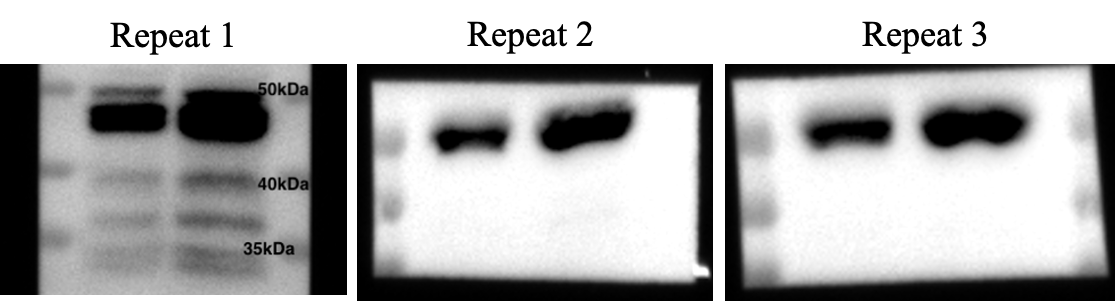


Corresponding to Figure 7L (HCT116β-actin).

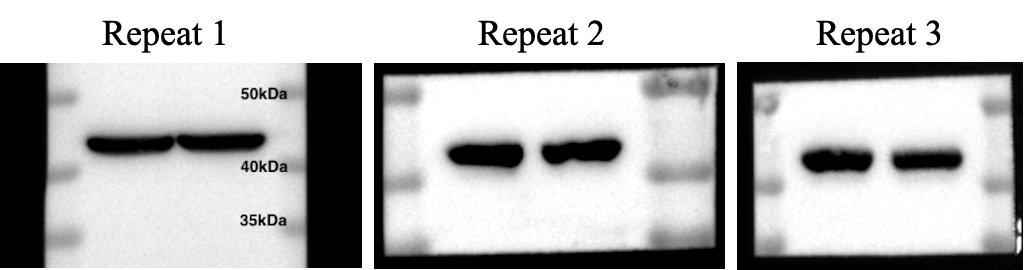


Corresponding to Figure 7L (SW480 GPX4).

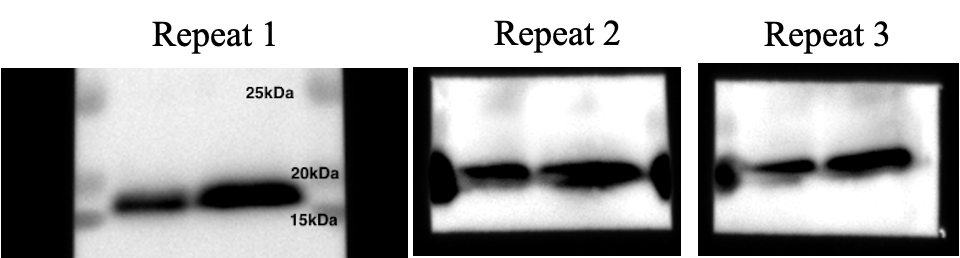


Corresponding to Figure 7L (SW480 TCF1).

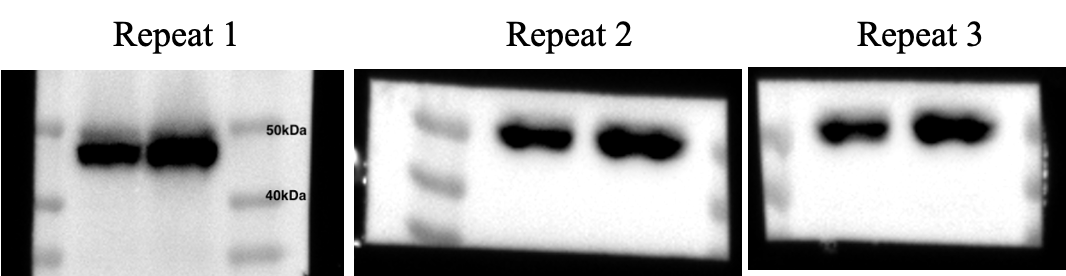


Corresponding to Figure 7L (SW480β-actin).

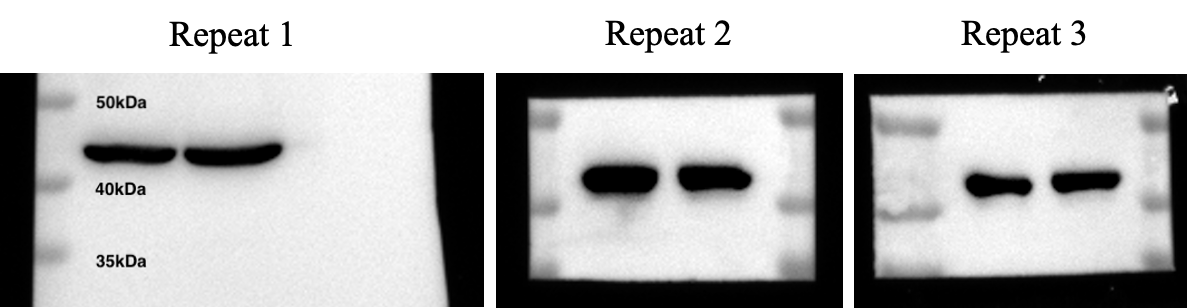


Corresponding to Figure 7P (LRP2).

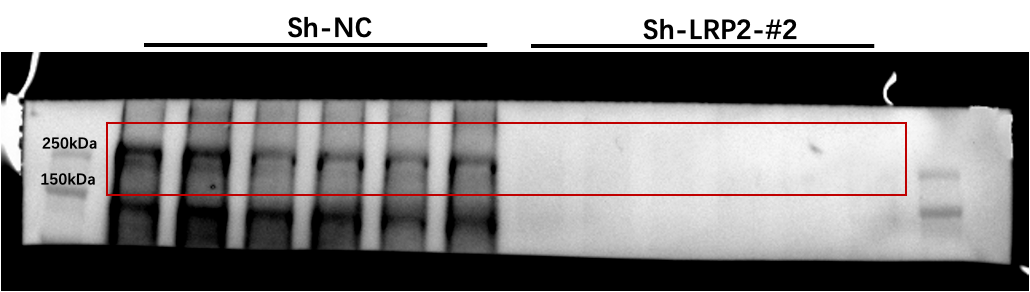


Corresponding to Figure 7P (β-catenin).

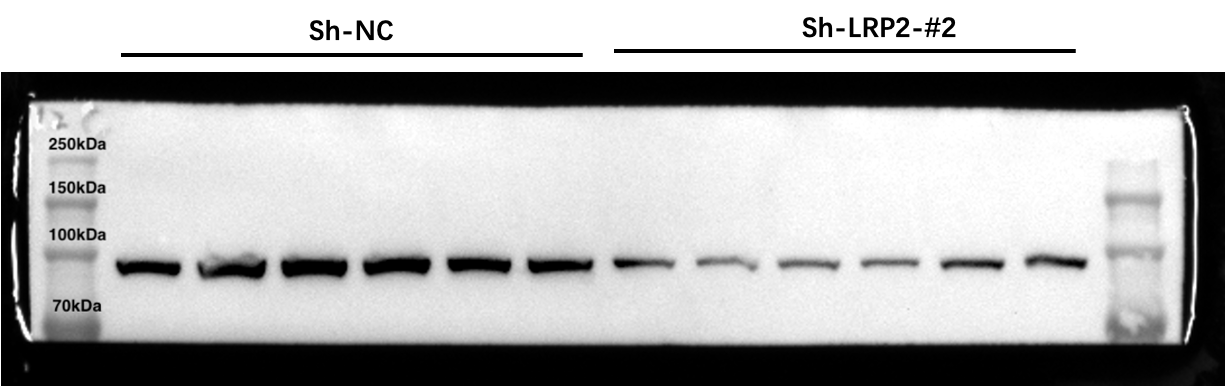


Corresponding to Figure 7P (pGSK-3β).

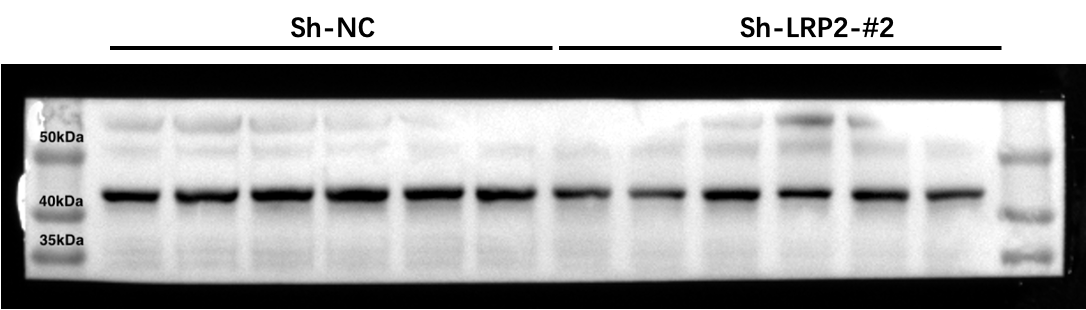


Corresponding to Figure 7P (GSK-3β).

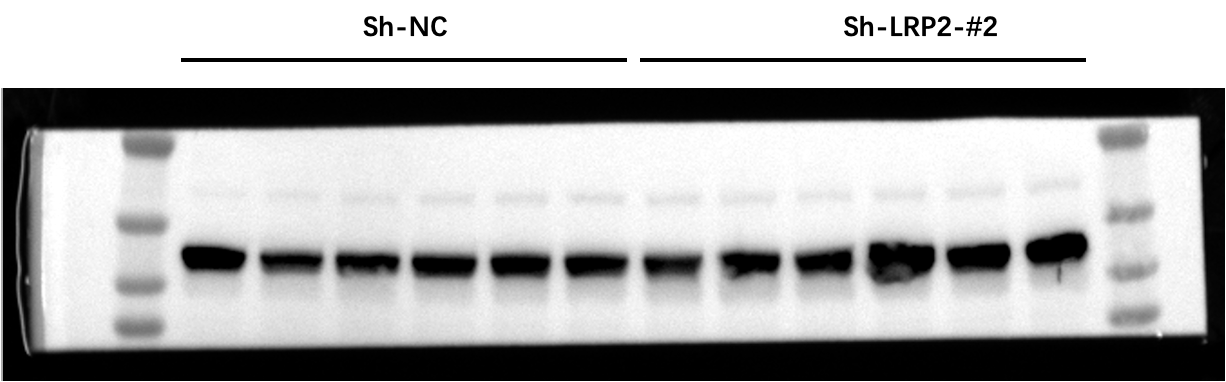


Corresponding to Figure 7P (TCF1).

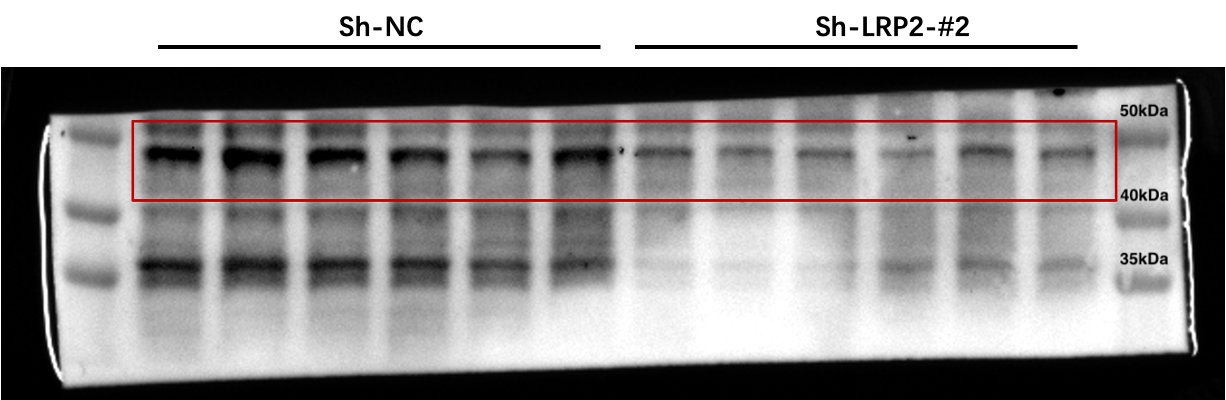


Corresponding to Figure 7P (GPX4).

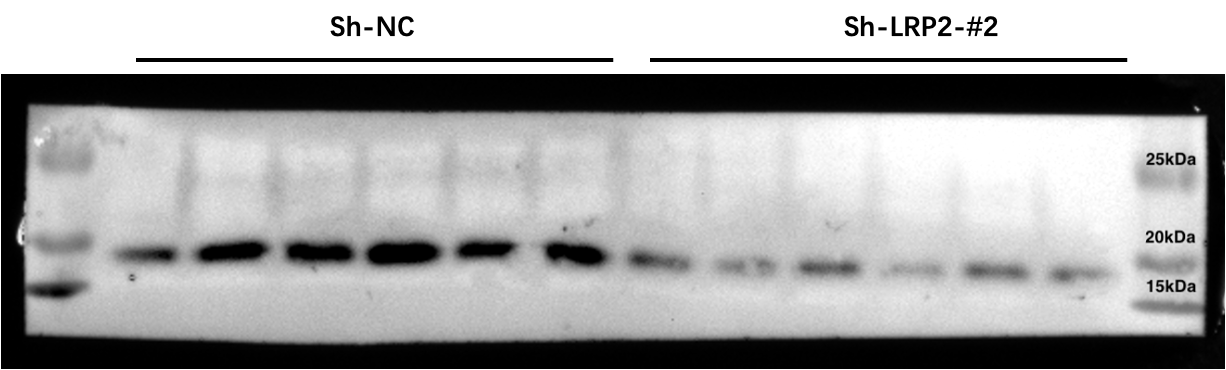


Corresponding to Figure 7P (β-actin).

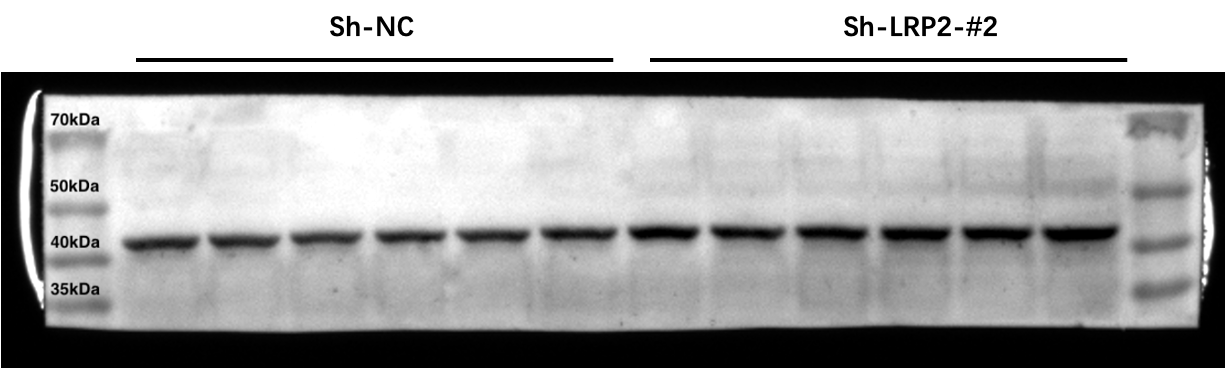


Corresponding to Figure 7Q (LRP2).

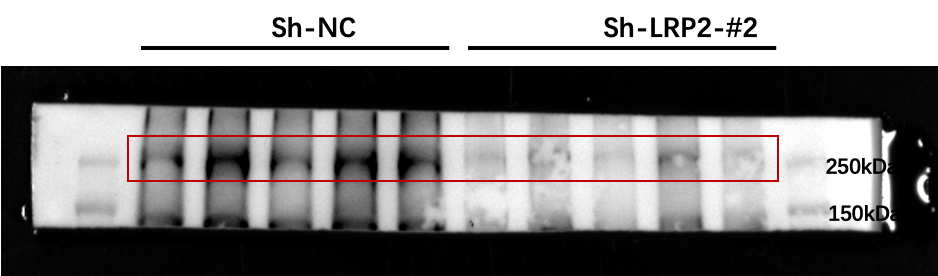


Corresponding to Figure 7Q (β-catenin).

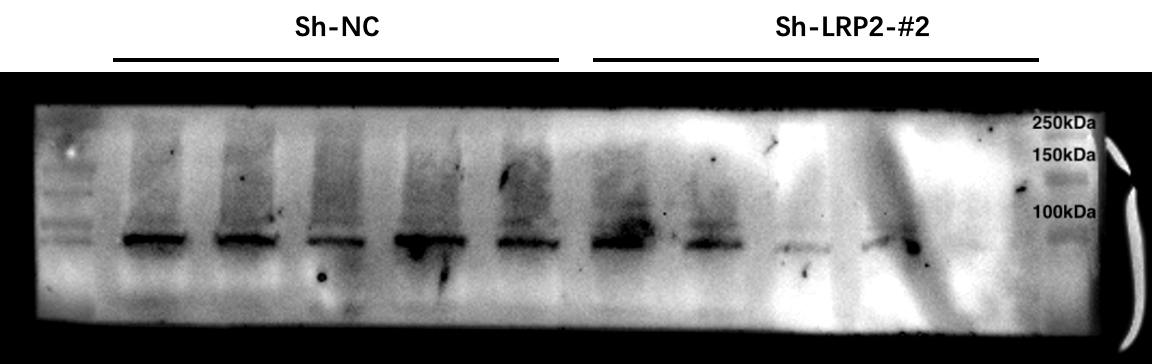


Corresponding to Figure 7Q (pGSK-3β).

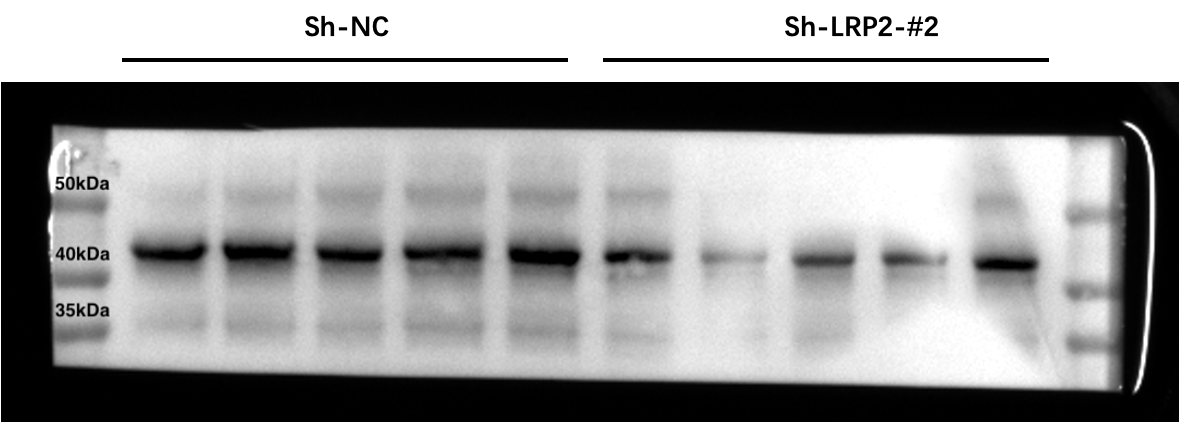


Corresponding to Figure 7Q (GSK-3β).

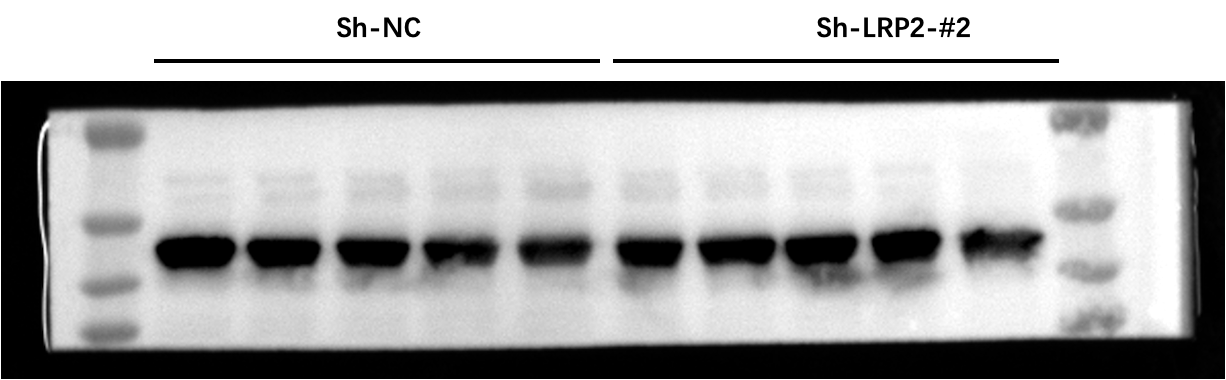


Corresponding to Figure 7Q (TCF1).

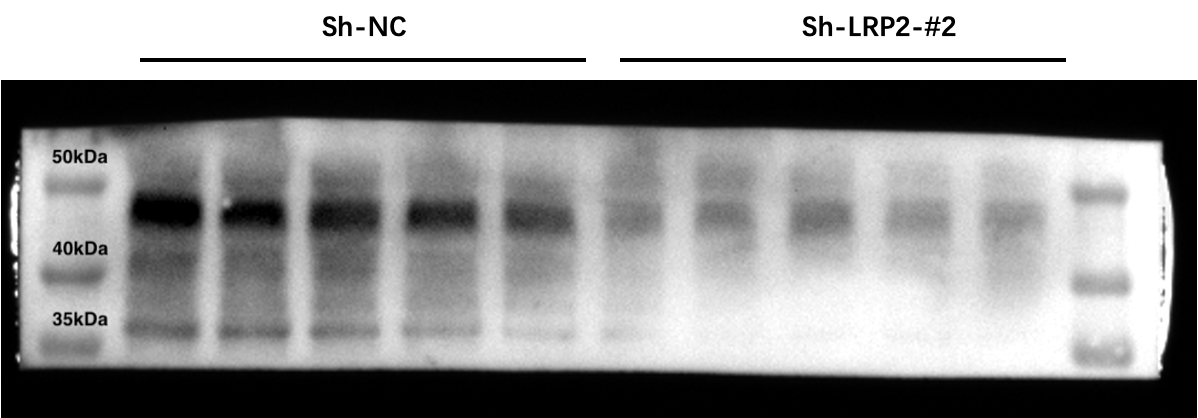


Corresponding to Figure 7Q (GPX4).

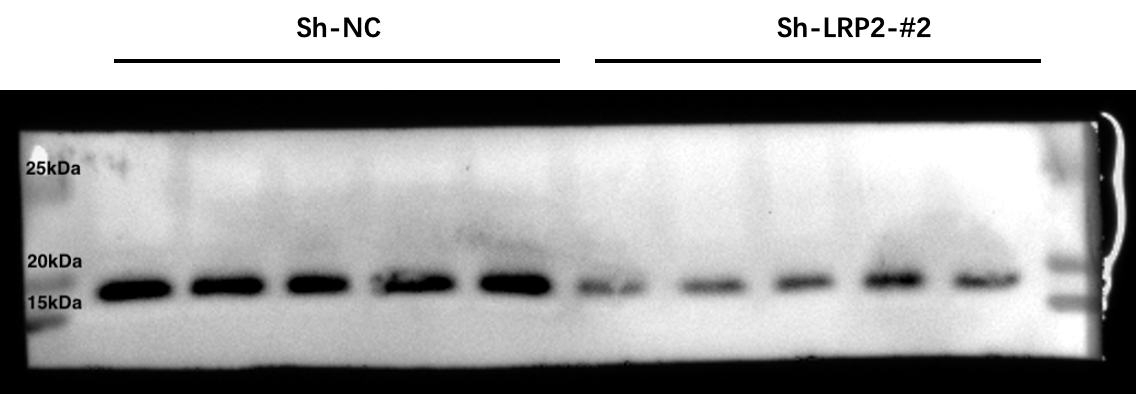


Corresponding to Figure 7Q (β-actin).

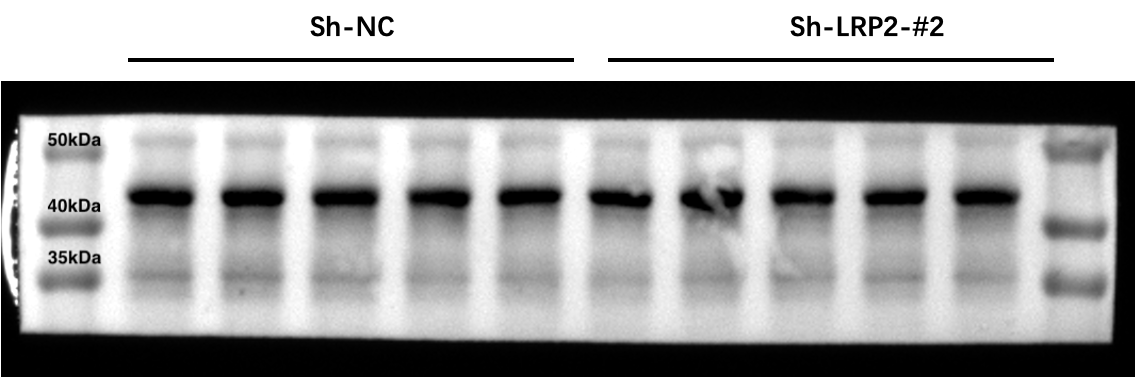


Corresponding to Supplementary Figure 2A (HCT116 and SW480, LRP2).

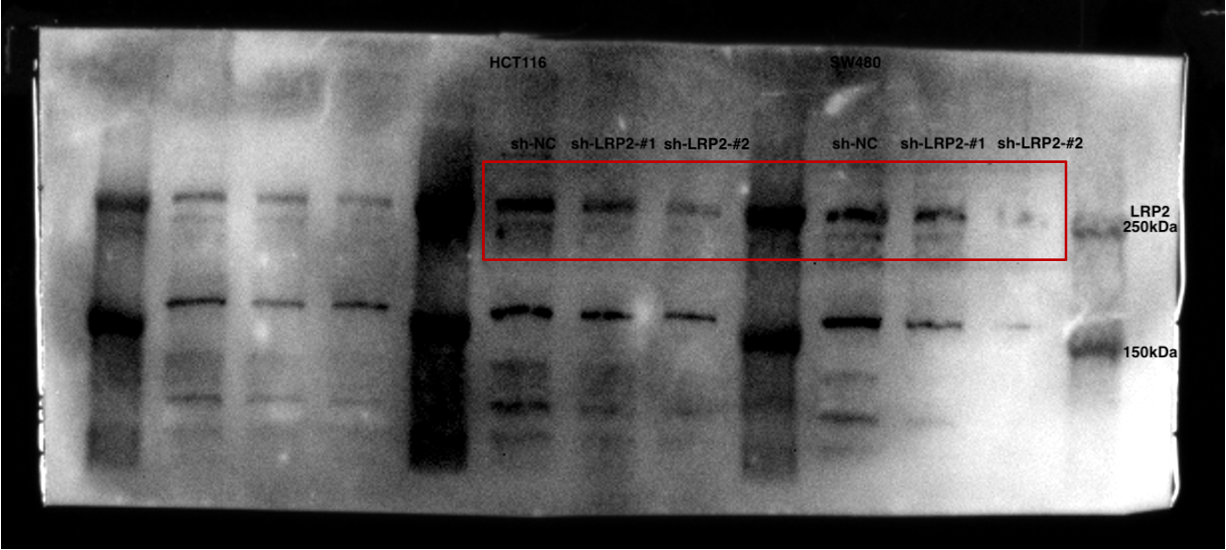


Corresponding to Supplementary Figure 2A (HCT116β-actin).

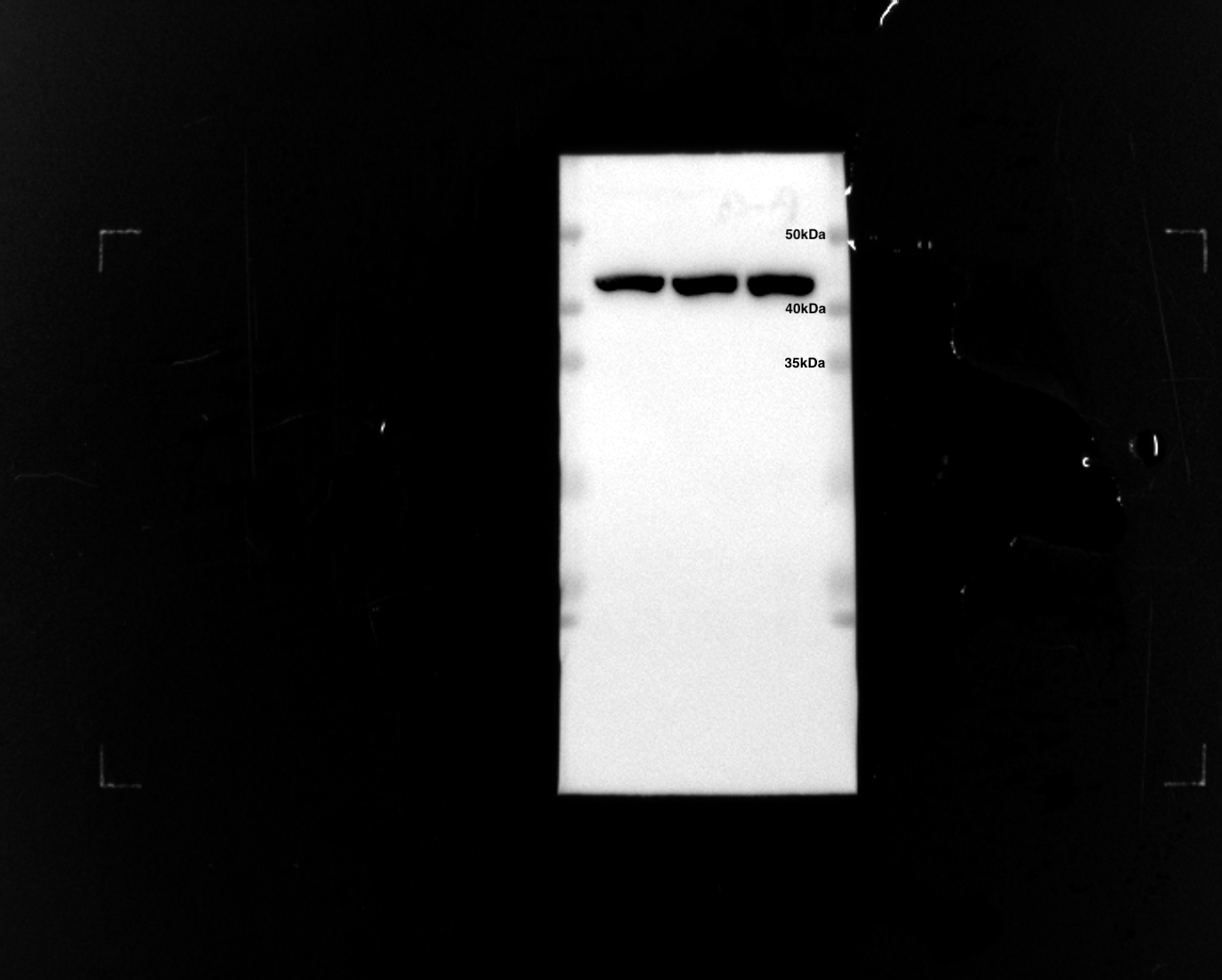


Corresponding to Supplementary Figure 2A (SW480β-actin).

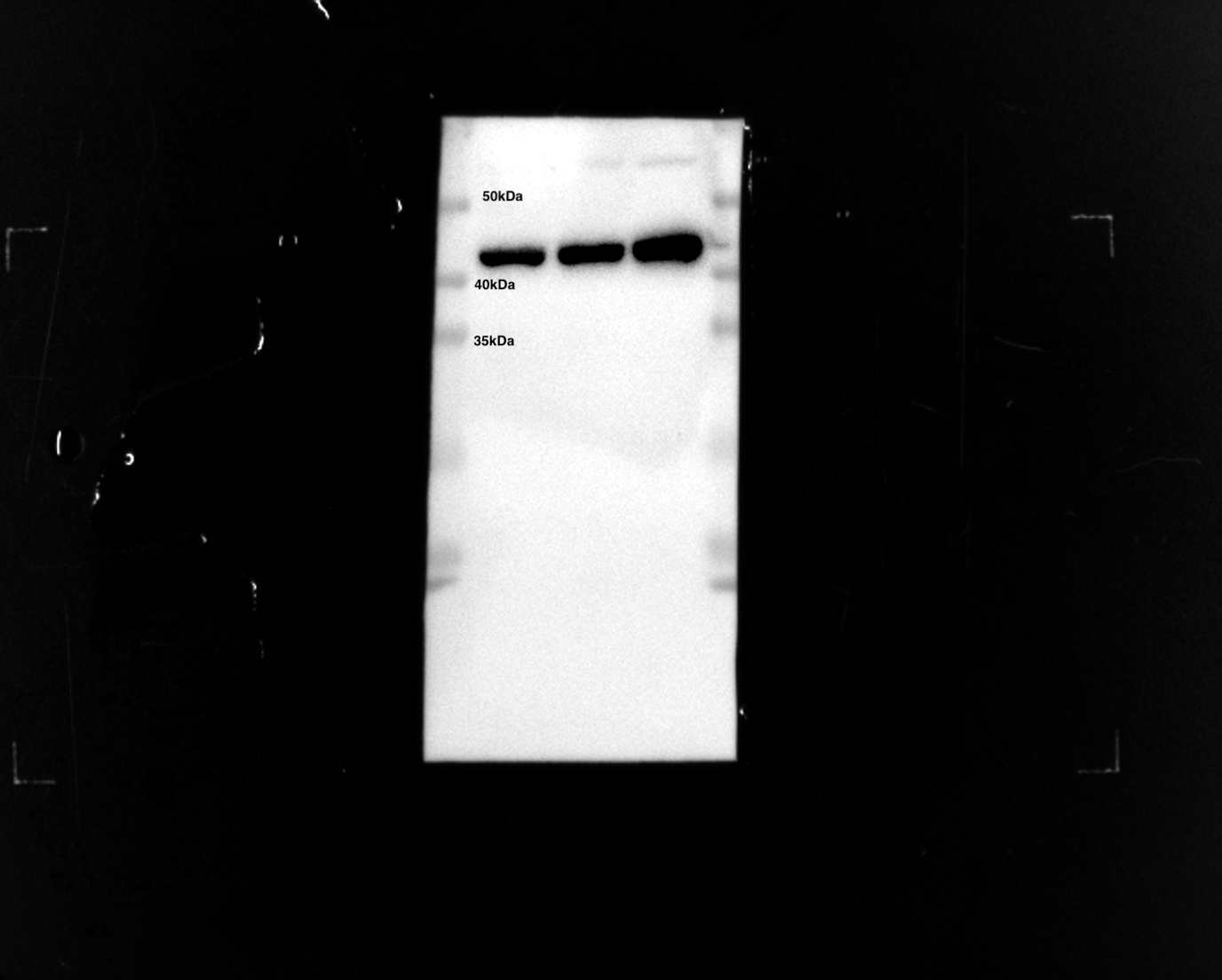


Corresponding to Supplementary Figure 3C (HCT116 GPX4).

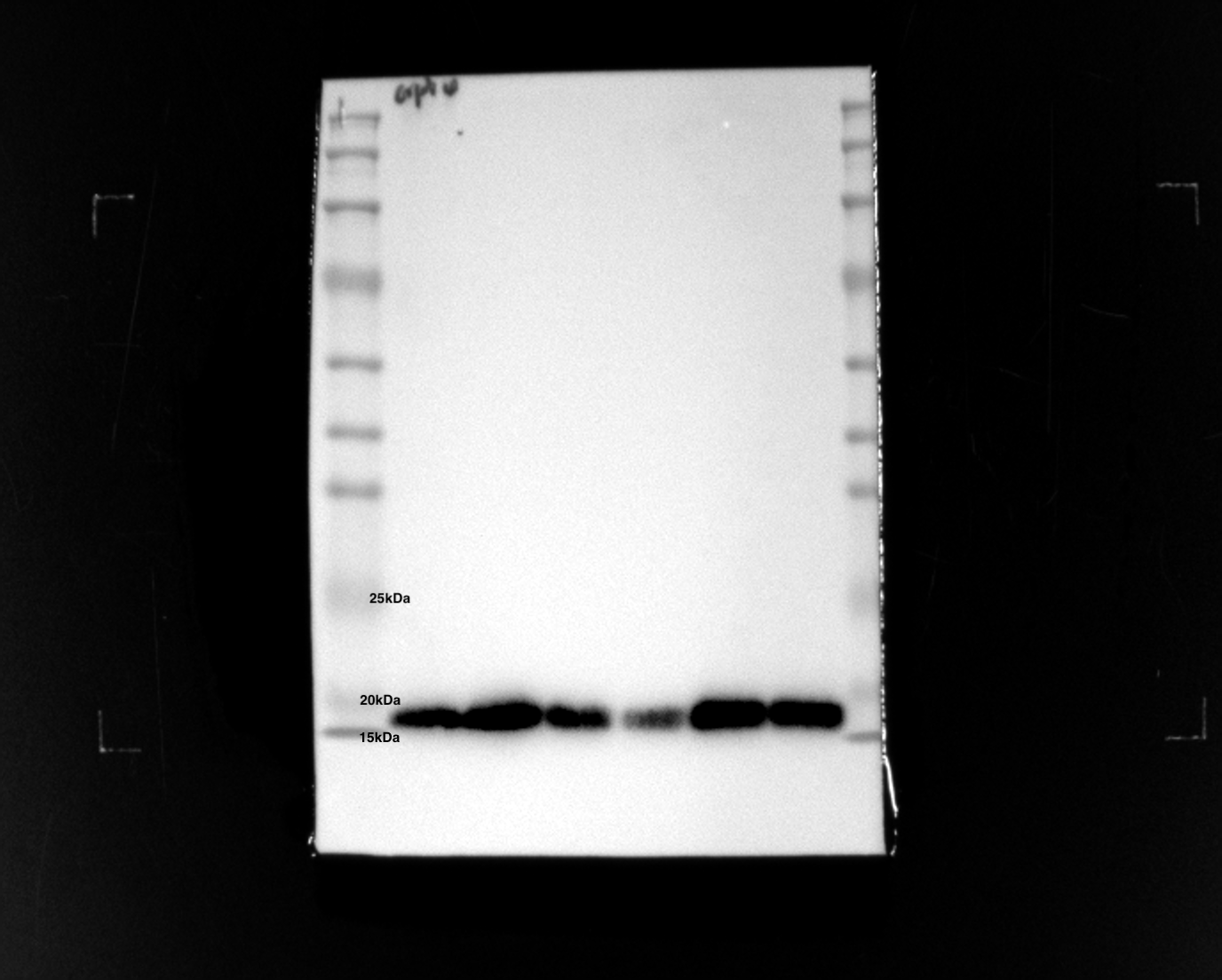


Corresponding to Supplementary Figure 3C (HCT116β-actin).

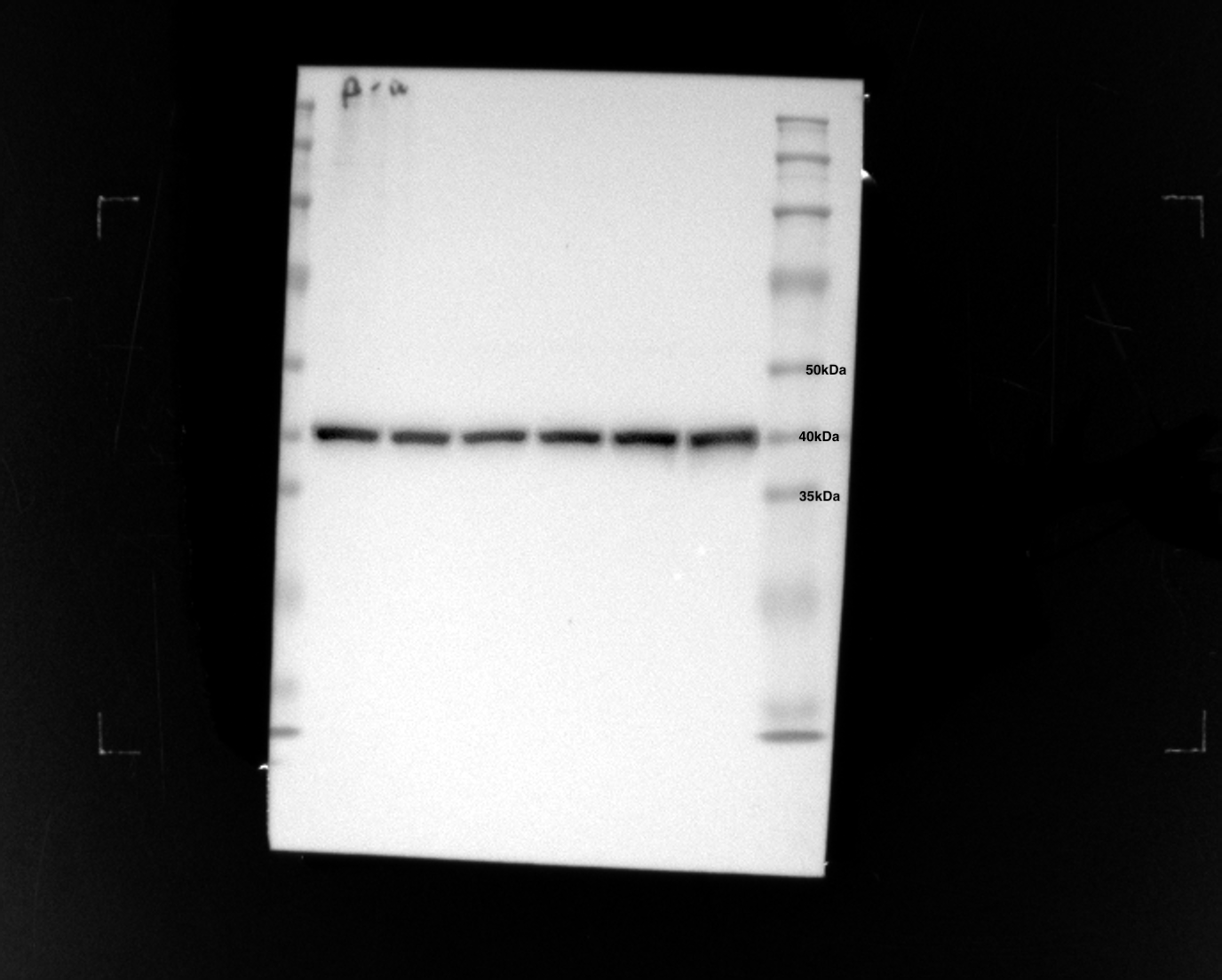


Corresponding to Supplementary Figure 3C (SW480 GPX4).

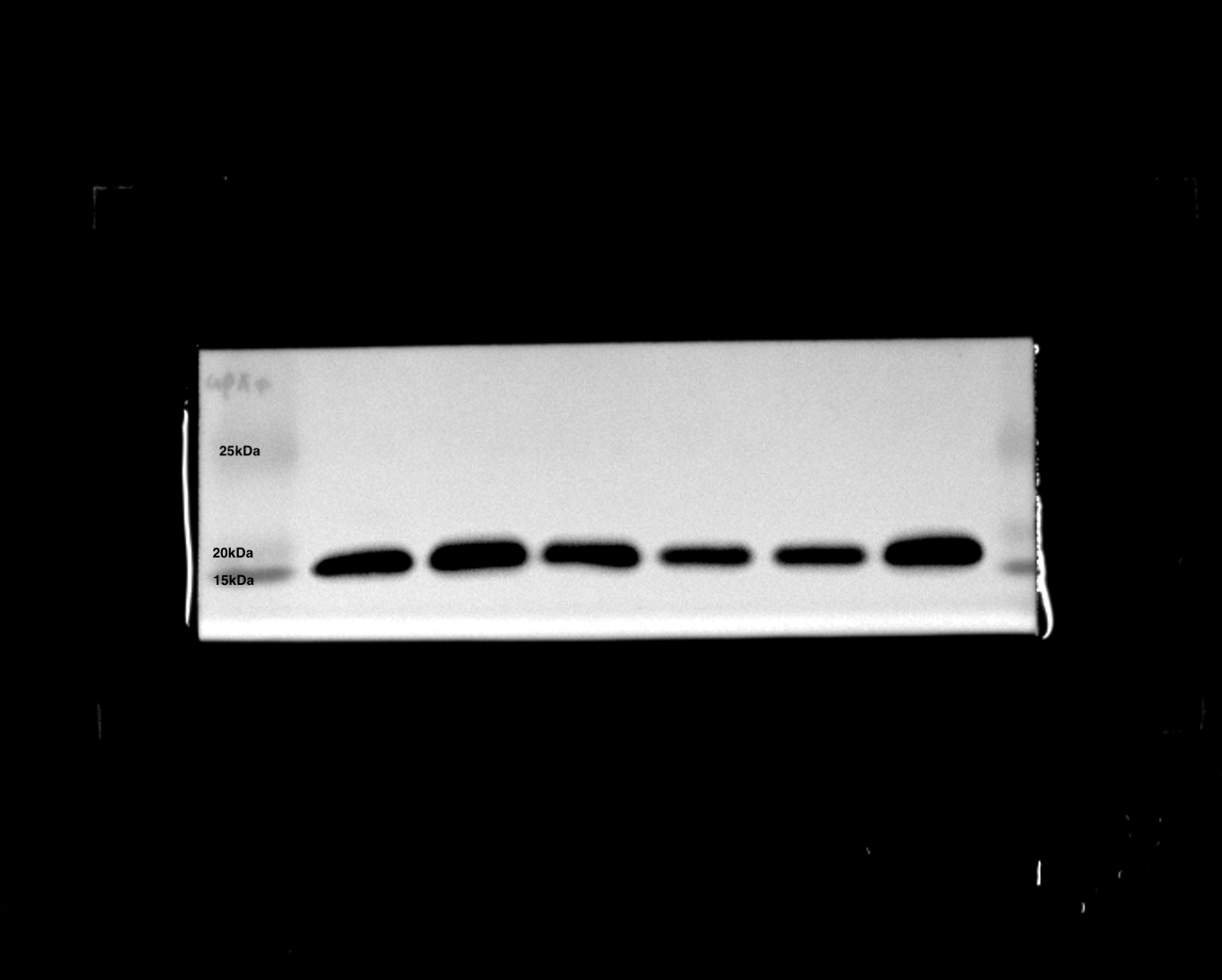


Corresponding to Supplementary Figure 3C (SW480β-actin).

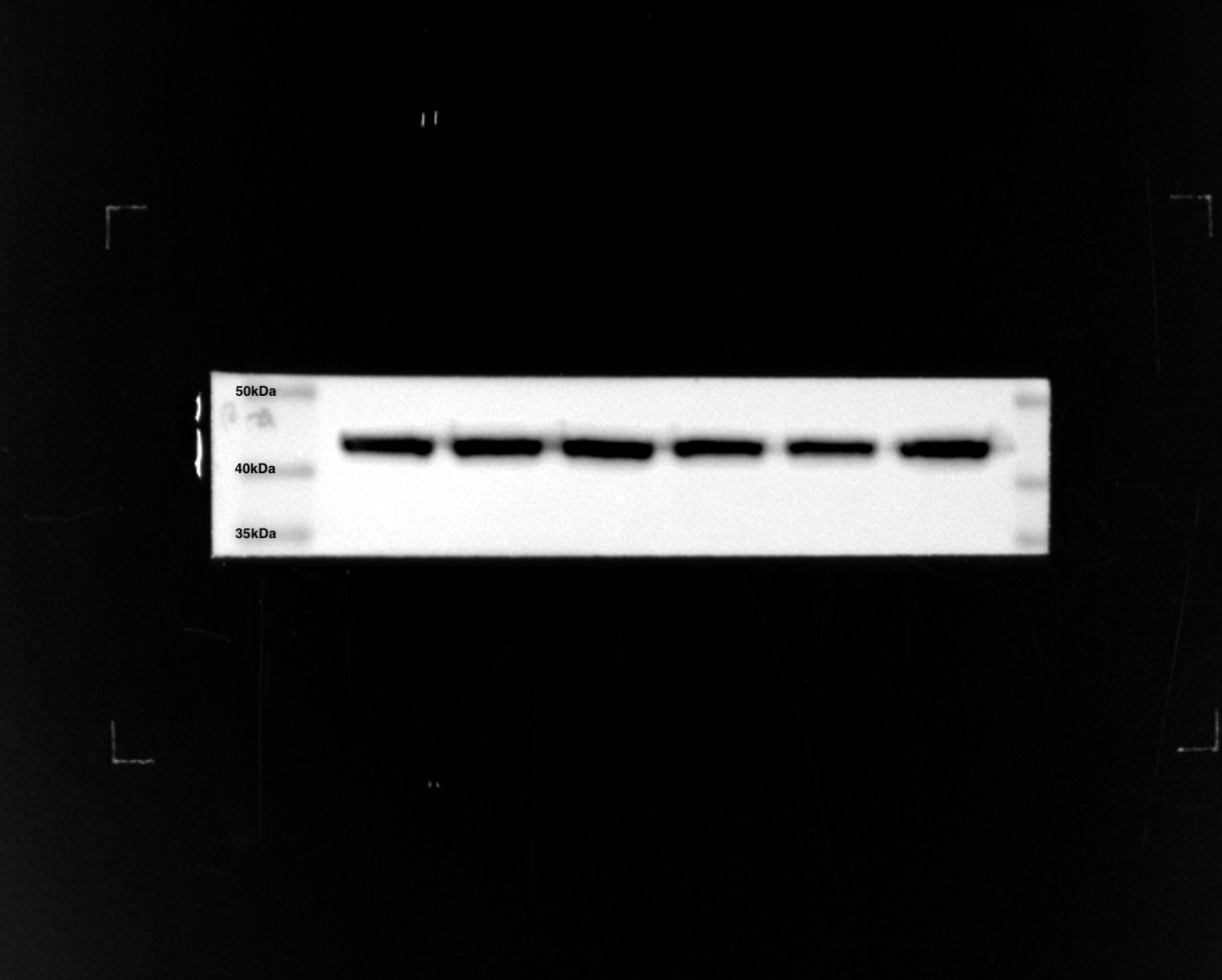


Corresponding to Supplementary Figure 4A (HCT116, TCF4).

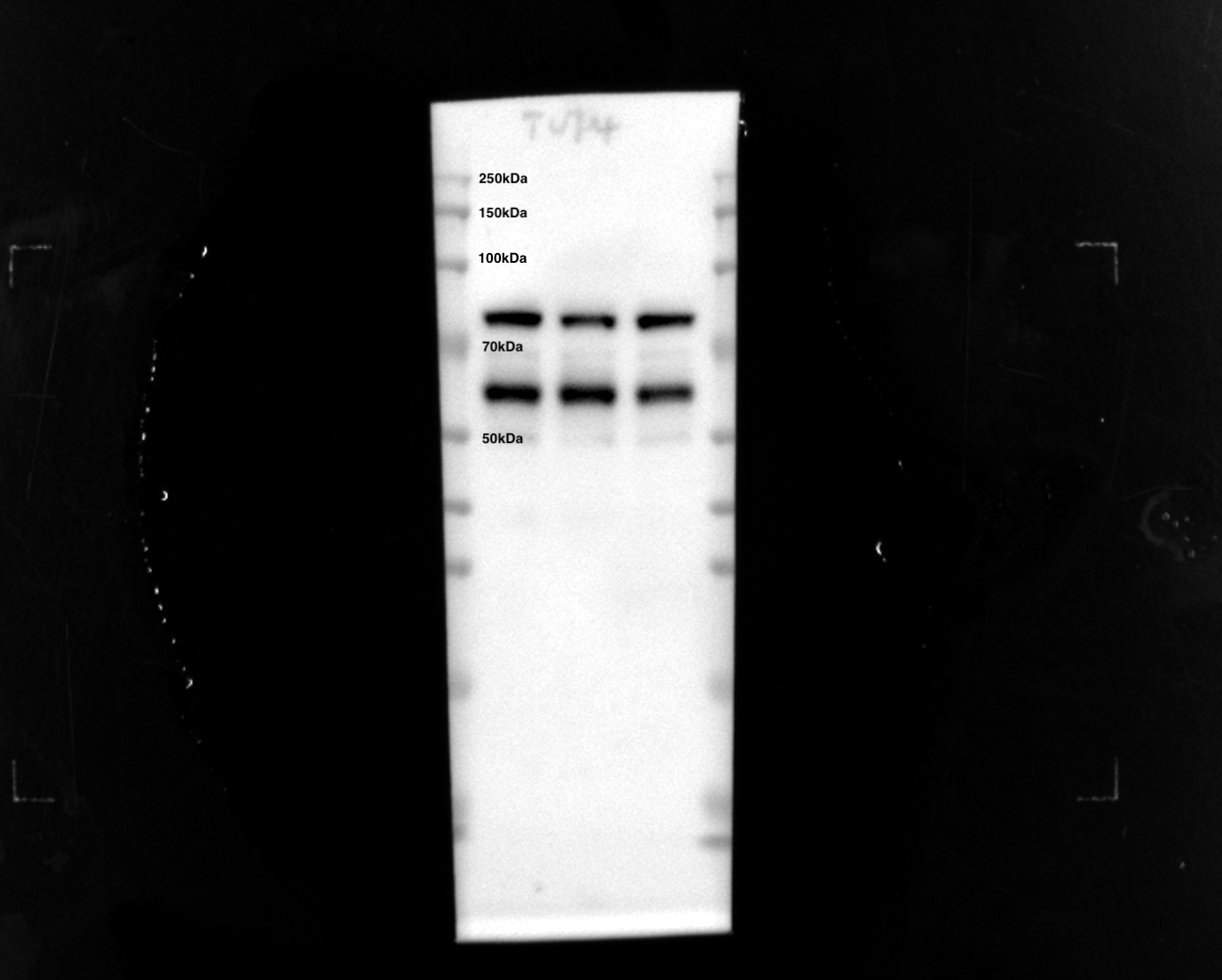


Corresponding to Supplementary Figure 4A (SW480, TCF4).

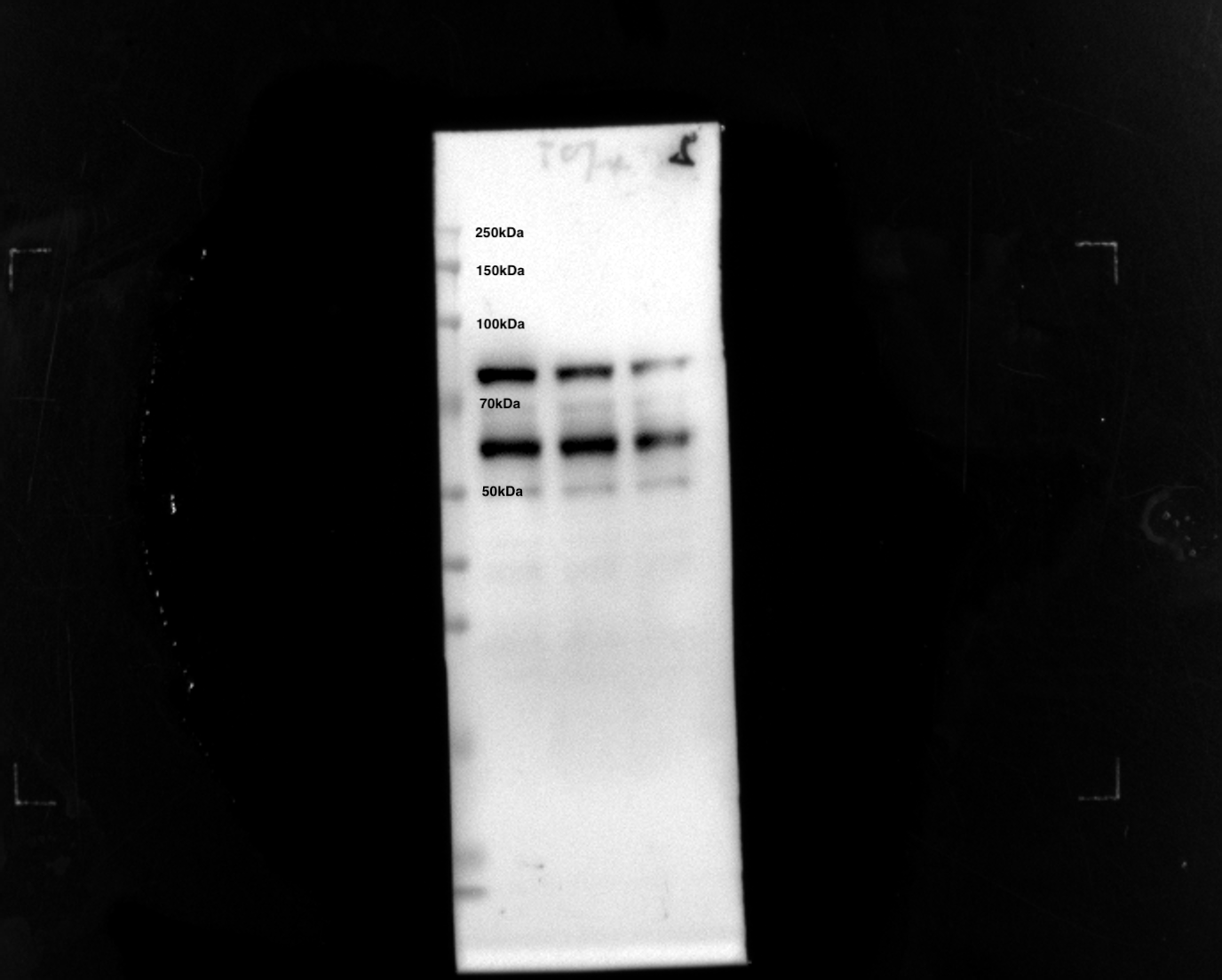

Supplement: Supplementary file 6 — Full-length uncropped Western blots [file 41420_2026_3161_MOESM6_ESM.docx]
